# Two-Temperature and Thermal Plasma Kinetic Theories

**DOI:** 10.3390/e28030275

**Published:** 2026-03-01

**Authors:** Vincent Giovangigli

**Affiliations:** Centre de Mathématiques Appliquées (CMAP), Centre National de la Recherche Scientifique (CNRS), École Polytechnique, 91128 Palaiseau, France; vincent.giovangigli@polytechnique.edu

**Keywords:** kinetic theory, plasma, Chapman-Enskog, magnetized, two-temperature, thermal, second-order correctors

## Abstract

The main objective of this paper is to establish links between two different plasma kinetic theories. We start by summarizing a two-temperature kinetic theory of multicomponent magnetized reactive plasmas, where electrons and heavy species have their own temperature. The Knudsen number is taken to be proportional to the square root of the mass ratio, and polyatomic species are taken into account. We next summarize the one-temperature kinetic theory of multicomponent magnetized reactive plasmas when the mass ratio remains of order unity. The complex tensorial structure of the transport fluxes is addressed, as well as the symmetry properties of the multicomponent transport coefficients. We then establish new links between these two theories by using the two-temperature scaling in the transport linear system obtained from the one-temperature kinetic theory. By expanding the solutions of the transport linear systems in terms of the Knudsen number, the flux structure of the two-temperature theory is recovered from the one-temperature theory as well as the second-order corrector terms. We also address the solution of transport linear systems by using fast and convergent iterative algorithms and their improvement for ionized mixtures.

## 1. Introduction

Ionized magnetized reactive gas mixtures have many practical applications, like plasma-enhanced chemical vapor deposition [1] or entry into the planetary atmosphere [2,3,4,5]. This is a strong motivation for investigating the kinetic theories of low temperature plasmas and the associated sound derivation of macroscopic plasma equations. Important ingredients in these kinetic theories are the presence of polyatomic species and the smallness of the mass ratio between electrons and heavier species.

The most general thermodynamic nonequilibrium models are the state-to-state models, where each internal state of a molecule is independent and considered as a separate species. Such state to state models, that may take into account electronic exited states as well as ionized species, have notably been investigated by Aliat, Kustova and Chikaoui [6], Bruno et al. [7], Capitelli et al. [8], Kustova and Puzyreva [9], Laux, Pierrot and Gessman [10], and Istomin and Kustova [11]. When there are partial equilibria between some of these internal states, it becomes possible to define species internal energy temperatures, and this reduces the complexity of the model, as investigated by Nagnibada and Kustova [3] and Colonna et al. [12]. When heavy species internal temperatures and translational temperatures are all equal, but differ from the electron temperature, a two-temperature plasma is then obtained, as investigated by Braginsky [13,14], Devoto [15], Chmielski and Ferziger [16], Daybelge [17], Petit and Darrozes [18], Kolesnikov and Tirsky [19], Degond and Lucquin [20], Magin and Degrez [21], Graille, Magin and Massot [22], and Orlac’h, Giovangigli, Novikova, and Roca y Cabarrocas [23]. Thermal plasmas are finally reached when the electron and heavy species temperatures coincide, and have been notably studied by Ludwig and Heil [2], Chapman and Cowling [24], Clemmow and Dougherty [25], Ferziger and Kaper [26], Rozhansky and Tsendin [27] and Giovangigli and Graille [28,29].

Application of the Chapman–Enskog theory to partially ionized mixtures of monatomic gases in the presence of electric and magnetic fields has been discussed by Chapman and Cowling [24] and Ferziger and Kaper [26] in a regime where there is only one temperature. These studies have been extended to the situation of partially ionized reactive polyatomic gas mixtures by Giovangigli and Graille [28,29]. Mixtures of monatomic gases at thermodynamic nonequilibrium with multitemperature transport have been investigated by Braginsky [13,14], Devoto [15], Chmieleski and Ferziger [16], Kolesnikov and Tirsky [19] and Magin and Degrez [21]. Convergence properties of the Chapman–Enskog expansion for transport coefficients of magnetized argon plasmas have been investigated by Bruno et al. [30,31,32]. Petit and Darrozes [18] have also established that a sound scaling is obtained when the Knudsen number is taken to be proportional to the square root of the electron-to-heavy-particle mass ratio. Degond and Lucquin [20] have investigated the corresponding multitime dynamics for a binary mixture of monatomic species. A comprehensive kinetic theory of two-temperature magnetized reactive multicomponent mixtures of monatomic species was then obtained by Graille, Magin and Massot [22] using the Petit and Darrozes scaling. The fast collision operators have been expanded in terms of the small parameter and the multiple dynamics of relaxation investigated, and the second-order corrector terms have been recovered. Next, this kinetic theory was then extended to the situation of polyatomic species by Orlac’h, Giovangigli, Novikova and Roca y Cabarrocas [23]. The Grad method has also been used for plasmas by Zhdanov et al. [33,34] and Magin, Martins and Torrilhon [35], as well as the moment method by Laguna and Pichard [36].

We first summarize the two-temperature kinetic theory for reactive multicomponent magnetized plasmas, taking into account the presence of polyatomic species [23]. The Knudsen number ϵ is taken to be proportional to the square root of the mass ratio, following the Petit and Darrozes scaling, so that the thermal velocity of the heavy species is of order O(1) and that of the electrons of order O(1/ϵ). Multiscale asymptotic expansions are performed and successively lead to the thermalization of electrons, the thermalization of heavy species, the equations for the electrons’ first-order perturbed distribution, the equations for the heavy species’ first-order perturbed distribution, the equations for the electrons’ second-order perturbed distribution and finally, to the fluid macroscopic equations. The rescaled Boltzmann equations, the linearized kinetic equations, the first-order fluid equations, and the structure of the transport linear systems, as well as convergent iterative algorithms, are reviewed.

We next investigate the situation of one-temperature multicomponent reactive magnetized plasmas for a mixture of polyatomic species when the mass ratio remains of order O(1) [28,29]. All the species thermal velocities are then O(1), and there is magnetization for heavy molecules. As a result, multicomponent diffusion fluxes are found to be non-isotropic, as well as the viscous tensor, and there are five shear viscosities. We review the rescaled Boltzmann equations, the linearized kinetic equations, the first-order fluid equations, and the structure of the real and complex transport linear systems, as well as convergent iterative algorithms. The linear systems associated with the first approximation of the multicomponent diffusion coefficients are often used in order to illustrate various results without the burden of heavy notation or cumbersome details.

The main aim of the paper is to finally establish new links between these two kinetic theories by using the small electron mass asymptotics within the transport linear systems of the one-temperature kinetic theory, in the situation where the electron and heavier species temperatures are equal. The structure of the two-temperature fluxes is notably recovered from the one-temperature theory by asymptotically expanding the solutions of the transport linear systems. The second-order corrector terms of the two-temperature kinetic theory are notably recovered from asymptotic expansions of the cross diffusion terms of the one-temperature theory. Since the goal of the paper is to establish new links between plasma kinetic theories, many complexities lay out of the scope of the present work, like radiation, surface effects, or quantum phenomena.

The two-temperature plasma kinetic theory is presented in Section 2, the thermal plasma kinetic theory in Section 3, and the new links between these kinetic theories are established in Section 4. A summary of notation is also presented in Appendix A.

## 2. Kinetic Theory of a Two-Temperature Plasma

We consider a mixture of monatomic and polyatomic gases interacting with chemical reactions and evolving in an electromagnetic field. A dimensional analysis is first summarized following the scaling introduced by Petit and Darrozes, where the Knudsen number ϵ is taken to be the square root of the electron-to-heavy-species mass ratio [18].

A multiscale Chapman–Enskog procedure is then performed and involves asymptotic expansions of the collision operators, the streaming operators, and the collision invariants in powers of ϵ [22,23]. The reference velocity must then be that of the heavy species $v_{h}$ for a consistent theory. An important difference with standard kinetic theories is that the thermal velocity of electrons is O(1/ϵ), whereas that of heavier species is O(1). The macroscopic equations and the transport fluxes at first order are then obtained by using scalar products with the rescaled collisional invariants [22,23].

### 2.1. Rescaled Boltzmann Equations

The state of the mixture is described by the species distribution functions denoted by $f_{i}\left(t,x,c_{i},i\right)$, where *i* is the index of the species, *t* the time, x the three-dimensional spatial coordinate, $c_{i}$ the velocity of the particles of the *i*th species, and *i* the index for the internal energy state of the *i*th species. The species indexing set is denoted by S={1,…,n}, where *n* is the number of species. We denote by $m_{i}$ the mass of the particles’ *i*th species, $q_{i}$ the particle charge of the *i*th species, $E_{ii}$ the internal energy of the *i*th species in the *i*th state, and $Q_{i}$ the indexing set of the quantum states i. The set H denotes the heavy species indexing set and e the electron so that we may also write S=H∪{e}. For a family of functions, $\xi_{i}$, i∈S; we use the compact notation $\xi ={\left(\xi_{i}\right)}_{i\in S}$, and similarly $\xi_{h}={\left(\xi_{i}\right)}_{i\in H}$, for a family of functions associated with the heavy species.

All species of the mixture have common reference quantities, except for some quantities where it is necessary to distinguish between electrons and the heavier species. The characteristic quantities are denoted with the ^★^ superscript, and when necessary, those for electrons with the e subscript and those for heavier species with the h subscript. Denoting by $m_{h}^{★}$ a reference mass for the heavy species and $m_{e}^{★}$ a reference mass for the electrons, the mass ratio $\epsilon ={\left(m_{e}^{★}/m_{h}^{★}\right)}^{1/2}$ is the key small parameter of the asymptotic analysis. The temperatures have a common scale $T^{★}=T_{h}^{★}=T_{e}^{★}$, as well as number densities $n^{★}=n_{h}^{★}=n_{e}^{★}$, fluid velocities $v^{★}=v_{h}^{★}=v_{e}^{★}$, elastic cross sections $\sigma^{★}=\sigma_{h}^{★}=\sigma_{e}^{★}$, and the free paths $l^{★}=1/n^{★}\sigma^{★}$. The characteristic thermal velocities of heavy species and electrons thus differ with $C_{h}^{★}={\left(k_{\;B}T^{★}/m_{h}^{★}\right)}^{1/2}$ and $C_{e}^{★}={\left(k_{\;B}T^{★}/m_{e}^{★}\right)}^{1/2}=C_{h}^{★}/\epsilon$, where $k_{\;B}$ denotes the Boltzmann constant. The Mach number is assumed to be of order unity with $v^{★}=C_{h}^{★}$. There are thus three different characteristic times, one for the electrons’ dynamics $t_{e}^{★}=l^{★}/C_{e}^{★}$, one for the heavy particles’ dynamics $t_{h}^{★}=l^{★}/C_{h}^{★}$, and one for the fluid dynamics $t^{★}=t_{h}^{★}/\epsilon$, so that $t_{e}^{★}=\epsilon t_{h}^{★}=\epsilon^{2}t^{★}$. The inelastic cross section must be of second-order for a two-temperature theory $\sigma_{eh}^{in}=\epsilon^{2}\sigma^{★}$, as established in [23]. The corresponding fluid length scale is $L^{★}=v^{★}t^{★}$ and the Knudsen number is given by $\mathrm{Kn}=l^{★}/L^{★}=\epsilon$. The electric field is such that the reference electrical and thermal energies are of the same order of magnitude, $q^{★}E^{★}L^{★}=k_{\;B}T^{★}$, and the magnetic field is estimated with the Hall numbers of electrons and heavy particles $\beta_{e}=q^{★}B^{★}t_{e}^{★}/m_{e}^{★}=1$ and $\beta_{h}=q^{★}B^{★}t_{h}^{★}/m_{h}^{★}=\epsilon$. The chemistry production terms are finally assumed to be of order O(ϵ). All the unknowns and independent variables may then be rescaled as well as the governing equations by using the characteristic quantities. Only the rescaled quantities and the rescaled equations are used in the following by using the same symbols as the original unscaled quantities for the sake of notational simplicity.

The rescaled Boltzmann equations of the two-temperature plasma are found in the form [22,23]:

(1) $$\begin{array}{c} \partial_{t}f_{e}+\frac{1}{\epsilon }c_{e}\cdot \nabla f_{e}+\frac{1}{\epsilon }\frac{q_{e}}{m_{e}}E^{\prime }\cdot \;\nabla_{\;\;c_{e}}f_{e}+\frac{1}{\epsilon^{2}}\frac{q_{e}}{m_{e}}\left(C_{e}\wedge B\right)\cdot \nabla_{\;\;c_{e}}f_{e}=\frac{1}{\epsilon^{2}}\left(\sum_{j\in H}J_{ej}+J_{ee}\right)+\epsilon \;C_{e}, \end{array}$$

(2) $$\begin{array}{c} \partial_{t}f_{i}+c_{i}\cdot \nabla f_{i}+\frac{q_{i}}{m_{i}}E^{\prime }\cdot \nabla_{\;\;c_{k}}f_{i}+\frac{q_{i}}{m_{i}}\left(C_{i}\wedge B\right)\cdot \nabla_{\;\;c_{k}}f_{i}=\frac{1}{\epsilon }\left(\sum_{j\in H}J_{ij}+\frac{1}{\epsilon }J_{ie}\right)+\epsilon \;C_{i},\;i\in H, \end{array}$$

where $E^{\prime }=E+v_{h}\wedge B$ denotes the electric field in the heavy species reference frame, E the electric field, B the magnetic field, $C_{e}=c_{e}-v_{h}$ the electron thermal speed, $C_{i}=c_{i}-v_{h}$, i∈H, the thermal speed of the particles of the *i*th species, $J_{ij}$, i,j∈H∪{e} the scattering collision operators, and $C_{i}$, i∈S the reactive collision operators. These collision operators are detailed in the literature and the details are omitted [3,4,22,23,37]. These operators notably involve nonreactive collisions between neutral particles that may be polarizable, between neutral and ionized, and between ionized, as well as reactive collisions. The reference velocity is that of the heavy species $v_{h}$ for a consistent theory. The different dynamics between the electrons and the heavy species then lead to successive solutions of the kinetic equations, starting with electrons and then considering the heavy species.

### 2.2. Chapman–Enskog Expansion

We introduce the electron partial bracket product

(3) $${\langle \;\langle \xi_{e},\zeta_{e}\rangle \;\rangle }_{e}=\int \xi_{e}⊙{\bar{\zeta }}_{e}dC_{\;e}$$

between two tensors $\xi_{e}$ and $\zeta_{e}$ that are functions of $C_{\;e}$, and that may have complex components, where ⊙ denotes the contracted product and ${\bar{\zeta }}_{e}$ the complex conjugate of $\zeta_{e}$. Similarly, we introduce the heavy species partial bracket product

(4) $${\langle \;\langle \xi_{h},\zeta_{h}\rangle \;\rangle }_{h}=\sum_{j\in H}\sum_{i\in Q_{i}}\int \xi_{i}⊙{\bar{\zeta }}_{i}dC_{\;i},$$

between the two families $\xi_{h}={\left(\xi_{i}\right)}_{i\in H}$ and $\zeta_{h}={\left(\zeta_{i}\right)}_{i\in H}$, where $\xi_{i}$ and $\zeta_{i}$ are tensors that depend on $C_{\;i}=c_{i}-v_{h}$ and *i*.

The collision invariants are decomposed in the form $\psi^{l}={\left(\psi_{e}^{l},\psi_{h}^{l}\right)}^{t}$. The electron invariant components are given by $\psi_{e}^{l}=\delta_{le}$ for l∈S, $\psi_{e}^{n+\nu }=\epsilon m_{e}C_{e\nu }$ for ν=1,2,3, $\psi_{e}^{n+4}=\frac{1}{2}m_{e}{\left|C_{e}\right|}^{2}$, and the heavy species invariant components are given by $\psi_{h}^{l}={\left(\delta_{li}\right)}_{i\in H}$ for l∈H, $\psi_{h}^{n+\nu }={\left(m_{i}C_{i\nu }\right)}_{i\in H}$ for ν=1,2,3, and $\psi_{h}^{n_{h}+4}={\left(\frac{1}{2}m_{i}c_{i}\cdot \;c_{i}+E_{iI}\right)}_{i\in H}$ where $C_{i\nu }$ is the component of the thermal velocity $C_{i}$ in the *v*th spatial coordinate. In particular, in the limit ϵ→0, there is no collisional invariant associated with electron momentum anymore.

The electron and heavy species distribution functions are expanded in the form

(5) $$f_{e}=f_{e}^{\left(0\right)}\left(1+\epsilon \phi_{e}^{\left(1\right)}+\epsilon \phi_{e}^{\left(2\right)}+O\left(\epsilon^{3}\right)\right),$$

(6) $$f_{i}=f_{i}^{\left(0\right)}\left(1+\epsilon \phi_{i}^{\left(1\right)}+O\left(\epsilon^{2}\right)\right),\;i\in H,$$

and the Enskog constraints are written separately for electrons with $\psi_{e}^{e}$ and $\psi_{e}^{n+4}$ and for the heavy species with $\psi_{h}^{l}$, l∈H, $\psi_{h}^{n+\nu }$, ν∈{1,2,3}, and $\psi_{h}^{n+4}$. The two-temperature kinetic theory has rich dynamics, and application of the Chapman–Enskog procedure first leads to the thermalization of electrons, and then to the thermalization of the heavy species [22,23].

The zeroth-order distributions are found to be Maxwellians for the electrons

(7) $$f_{e}^{\left(0\right)}=n_{e}{\left(\frac{m_{e}}{2\pi k_{\;B}T_{e}}\right)}^{\frac{3}{2}}\exp\left\{-\frac{m_{e}}{2k_{\;B}T_{e}}{\left|c_{e}-v_{h}\right|}^{2}\right\},$$

where $m_{e}$ is the electron mass, $k_{\;B}$ the Boltzmann constant, $T_{e}$ the electron temperature, $n_{e}$ the electron number density, and $v_{h}$ the average heavy particle velocity, and next, for the heavy species,

(8) $$f_{i}^{\left(0\right)}=n_{i}{\left(\frac{m_{i}}{2\pi k_{\;B}T_{h}}\right)}^{\frac{3}{2}}\frac{a_{iI}}{Z_{i}^{\mathrm{int}}}\exp\left\{-\frac{m_{i}}{2k_{\;B}T_{h}}{\left|c_{i}-v_{h}\right|}^{2}-\frac{E_{iI}}{k_{\;B}T_{h}}\right\},\;i\in H,$$

where $n_{i}$ denotes the number density of the *i*th species, $m_{i}$ the mass of the particles of the *i*th species, $T_{h}$ the heavy species temperature, *E*_*i*i_ the internal energy of the *i*th species in the ith state, *a*_*i*i_ the degeneracy of the *i*th state, and $Z_{i}^{\mathrm{int}}=\sum_{I\in Q_{i}}a_{iI}\exp\left(-\frac{E_{iI}}{k_{\;B}T_{h}}\right)$ the partition function for the internal energy of the *i*th species. The zeroth-order equations for the electrons are found to be of parabolic type and include zeroth-order diffusive terms [22,23]. The zeroth-order equations for the heavy species are found to be the traditional hyperbolic Euler equations [22,23].

The thermodynamic properties may be evaluated from the Maxwellian distributions, and it is found that $p_{e}=n_{e}k_{\;B}T_{e}$, $E_{e}=n_{e}{\bar{E}}_{e}=n_{e}\frac{3}{2}k_{\;B}T_{e}$, $p_{h}=n_{h}k_{\;B}T_{h}$, $E_{h}=\sum_{i\in H}n_{i}{\bar{E}}_{i}$ where $n_{h}=\sum_{i\in H}n_{i}$, ${\bar{E}}_{i}=\frac{3}{2}k_{\;B}T_{h}+{\bar{E}}_{i}^{\mathrm{int}}$ and ${\bar{E}}_{i}^{\mathrm{int}}=\sum_{I\in Q_{i}}\frac{a_{iI}E_{iI}}{Z_{i}^{\mathrm{int}}}\exp\left(-\frac{E_{iI}}{k_{\;B}T_{h}}\right)$, as well as $p=p_{e}+p_{h}$ and $E=E_{e}+E_{h}$. Further introducing the translational energy partition function of the *i*th species per unit volume $Z_{i}^{\mathrm{tr}}={\left(2\pi m_{i}k_{\;B}T_{h}/{h_{P}}^{2}\right)}^{3/2}$ for i∈H and by $Z_{e}^{\mathrm{tr}}={\left(2\pi m_{e}k_{\;B}T_{e}/{h_{P}}^{2}\right)}^{3/2}$ for electrons, the complete partition function is given by $Z_{i}=Z_{i}^{\mathrm{tr}}Z_{i}^{\mathrm{int}}$ for all i∈S. The Gibbs function of the electrons per unit volume is then $G_{e}=k_{\;B}T_{e}\log\left(n_{e}/Z_{e}\right)$, and that of the heavier species $G_{i}=k_{\;B}T_{h}\log\left(n_{i}/Z_{i}\right)$ for i∈H. The corresponding entropy per unit volume then reads $S_{i}=\frac{5}{2}k_{\;B}+\frac{{\bar{E}}_{i}}{T_{h}}-k_{\;B}\log\left(n_{i}/Z_{i}\right)$ for i∈H, and that of electrons, $S_{e}=\frac{5}{2}k_{\;B}+\frac{{\bar{E}}_{e}}{T_{e}}-k_{\;B}\log\left(n_{e}/Z_{e}\right)$.

### 2.3. Linearized Kinetic Equations

Application of the Chapman–Enskog procedure then sequentially leads to linearized equations for the electron first-order perturbed distribution $\phi_{e}^{\left(1\right)}$, for the heavy species first-order perturbed distributions $\phi_{h}^{\left(1\right)}={\left(\phi_{i}^{\left(1\right)}\right)}_{i\in H}$, and finally for the electron second-order perturbed distribution $\phi_{e}^{\left(2\right)}$.

Denoting by $I_{e}$ the electron linearized collision operator, the equations governing $\phi_{e}^{\left(1\right)}$ are found in the form

(9) $$\left\{\begin{array}{c} I_{e}\left(\phi_{e}^{\left(1\right)}\right)+\frac{q_{e}}{m_{e}}\left(C_{e}\wedge B\right)\cdot \;\partial_{C_{e}}\phi_{e}^{\left(1\right)}=\psi_{e}^{\left(1\right)},\\ {\langle \;\langle f_{e}^{\left(0\right)}\phi_{e}^{\left(1\right)},\psi_{e}^{l}\rangle \;\rangle }_{e}=0,\;l\in \left\{e,n+4\right\}. \end{array}\right.$$

In the limit ϵ→0, there are only two collisional invariants for the electrons, namely the electron number $\psi_{e}^{e}$ and electron kinetic energy $\psi_{e}^{n+4}$. There is also a magnetization for electrons, since with scaled variables, we have $B^{★}=O\left(\epsilon \right)$ and $C^{★}=O\left(1/\epsilon \right)$. The right-hand side $\Psi_{e}$ may be decomposed in the form

(10) $$\psi_{e}^{\left(1\right)}=-\Psi_{e}^{D_{e}}\cdot \;\left(\nabla p_{e}-n_{e}q_{e}E^{\prime }\right)-\Psi_{e}^{{\hat{\lambda }}_{e}}\cdot \;\nabla \left(\frac{1}{k_{\;B}T_{e}}\right),$$

where $E^{\prime }=E+v_{h}\wedge B$ is the electric field in the heavy species reference frame and $p_{e}=n_{e}k_{\;B}T_{e}$ the electron partial pressure, and the expression of the coefficients $\Psi_{e}^{D_{e}}$ and $\Psi_{e}^{{\hat{\lambda }}_{e}}$ may be found in the literature [22,23]. The perturbed distribution $\phi_{e}^{\left(1\right)}$ is then in the form

$$\phi_{e}^{\left(1\right)}=-\Phi_{e}^{D_{e}}\cdot \;\left(\nabla p_{e}-n_{e}q_{e}E^{\prime }\right)-\Phi_{e}^{{\hat{\lambda }}_{e}}\cdot \;\nabla \left(\frac{1}{k_{\;B}T_{e}}\right),$$

and the vector coefficients $\Phi_{e}^{D_{e}}$ and $\Phi_{e}^{{\hat{\lambda }}_{e}}$ are solved by using integral equations with complex unknowns [22,23]. Keeping in mind that the operator $I_{e}$ is isotropic, so that it transforms any tensor product of $C_{\;e}$ into a similar tensor, the coefficient $\Phi_{e}^{D_{e}}$ is decomposed in the form

(11) $$\Phi_{e}^{D_{e}}=\phi_{i}^{D_{e}\left(1\right)}C_{\;e}+\phi_{i}^{D_{e}\left(2\right)}C_{\;e}\wedge B+\phi_{i}^{D_{e}\left(3\right)}C_{\;e}\cdot \;B\;B,$$

and a similar decomposition is introduced for $\Phi_{e}^{{\hat{\lambda }}_{e}}$. The tensorial decomposition (11) then allows transforming the differential–integral Equation (9) into an integral equation. The resulting integral equations are then solved by using complex numbers that conveniently take into account the rotation arising from the magnetic field. This method is illustrated in more detail in Section 3.3 and yields magnetized transport for electrons.

Denoting by $I_{h}={\left(I_{i}\right)}_{i\in H}$ the heavy species linearized collision operator, the equations governing $\phi_{h}^{\left(1\right)}={\left(\phi_{i}^{\left(1\right)}\right)}_{i\in H}$ are in the form

(12) $$\left\{\begin{array}{c} I_{i}\left(\phi_{h}^{\left(1\right)}\right)=\Psi_{i}^{\left(1\right)},\;\;i\in H,\\ {\langle \;\langle f_{h}^{\left(0\right)}\phi_{h}^{\left(1\right)},\psi_{h}^{l}\rangle \;\rangle }_{h}=0,\;l\in H\cup \left\{n+1,n+2,n+3,n+4\right\}. \end{array}\right.$$

There is no magnetization term in the heavy species integral equations, since for scaled variables, we have $B^{★}=O\left(\epsilon \right)$ and $C_{h}^{★}=O\left(1\right)$ for i∈H, in such a way that the vector product $C_{\;i}\wedge B$ is negligeable for i∈H. However, magnetization will arise from the coupling with the electrons and we need to introduce some notation in order to write the right-hand side. Letting B=B/|B|, we introduce for any vector x the associated vectors

$$x^{\|}=x\cdot \;B\;\;B,\;x^{⊥}=x-x^{\|},\;x^{⊙}=B\wedge x.$$

The vectors $x^{\|}$, $x^{⊥}$ and $x^{⊙}$ are mutually orthogonal and obtained from x by applying the linear operators $M^{\|}=B⊗B$, $M^{⊥}=I-B⊗B$ and $M^{⊙}=R\left(B\right)$, where R(B) is the rotation operator associated with the vector B so that R(B)x=B∧x. The right-hand side $\Psi_{h}^{\left(1\right)}={\left(\Psi_{i}^{\left(1\right)}\right)}_{i\in H}$ may then be decomposed in the form

(13) $$\begin{array}{cc} \Psi_{i}^{\left(1\right)}= & -\Psi_{i}^{\eta_{h}}\;:\;\nabla v_{h}-\frac{1}{3}\Psi_{i}^{\kappa_{h}}\;\nabla \cdot \;v_{h}-\sum_{j\in H}\Psi_{i}^{D_{j}}\cdot \;\left(\nabla p_{j}-n_{j}q_{j}E^{\prime }\right)-\Psi_{i}^{{\hat{\lambda }}_{h}}\cdot \;\nabla \left(\frac{1}{k_{\;B}T_{h}}\right)\\ \; & -\Psi_{i}^{D_{e}\|}\cdot \;{\left(\nabla p_{e}-n_{e}q_{e}E^{\prime }\right)}^{\|}-\Psi_{i}^{D_{e}⊥}\cdot \;{\left(\nabla p_{e}-n_{e}q_{e}E^{\prime }\right)}^{⊥}-\Psi_{i}^{D_{e}⊙}\cdot \;{\left(\nabla p_{e}-n_{e}q_{e}E^{\prime }\right)}^{⊙}\\ \; & -\Psi_{i}^{{\hat{\lambda }}_{e}\|}\cdot \;\nabla {\left(\frac{1}{k_{\;B}T_{e}}\right)}^{\|}-\Psi_{i}^{{\hat{\lambda }}_{e}⊥}\cdot \;\nabla {\left(\frac{1}{k_{\;B}T_{e}}\right)}^{⊥}-\Psi_{i}^{{\hat{\lambda }}_{e}⊙}\cdot \;\nabla {\left(\frac{1}{k_{\;B}T_{e}}\right)}^{⊙}-\Psi_{i}^{\Theta }\left(T_{e}-T_{h}\right), \end{array}$$

where $p_{j}$ denotes the partial pressure of the *j*th species, $n_{j}$ the number density of the *j*th species, and $q_{j}$ the charge of the particles of the *j*th species, and the expression of the coefficients $\Psi_{i}^{\eta_{h}}$, $\Psi_{i}^{\kappa_{h}}$, $\Psi_{i}^{D_{j}}$, $\Psi_{i}^{{\hat{\lambda }}_{h}}$ $\Psi_{i}^{D_{e}\|}$, $\Psi_{i}^{D_{e}⊥}$, $\Psi_{i}^{D_{e}⊙}$, $\Psi_{i}^{{\hat{\lambda }}_{e}\|}$, $\Psi_{i}^{{\hat{\lambda }}_{e}⊥}$, $\Psi_{i}^{{\hat{\lambda }}_{e}⊙}$ and $\Psi_{i}^{\Theta }$ may be found in the literature [22,23]. The heavy species perturbed distributions are expanded in a similar way

$$\begin{array}{cc} \phi_{i}^{\left(1\right)}= & -\Phi_{i}^{\eta_{h}}\;:\;\nabla v_{h}-\frac{1}{3}\Phi_{i}^{\kappa_{h}}\;\nabla \cdot \;v_{h}-\sum_{j\in H}\Phi_{i}^{D_{j}}\cdot \;\left(\nabla p_{j}-n_{j}q_{j}E^{\prime }\right)-\Phi_{i}^{{\hat{\lambda }}_{h}}\cdot \;\nabla \left(\frac{1}{k_{\;B}T_{h}}\right)\\ \; & -\Phi_{i}^{D_{e}\|}\cdot \;{\left(\nabla p_{e}-n_{e}q_{e}E^{\prime }\right)}^{\|}-\Phi_{i}^{D_{e}⊥}\cdot \;{\left(\nabla p_{e}-n_{e}q_{e}E^{\prime }\right)}^{⊥}-\Phi_{i}^{D_{e}⊙}\cdot \;{\left(\nabla p_{e}-n_{e}q_{e}E^{\prime }\right)}^{⊙}\\ \; & -\Phi_{i}^{{\hat{\lambda }}_{e}\|}\cdot \;\nabla {\left(\frac{1}{k_{\;B}T_{e}}\right)}^{\|}-\Phi_{i}^{{\hat{\lambda }}_{e}⊥}\cdot \;\nabla {\left(\frac{1}{k_{\;B}T_{e}}\right)}^{⊥}-\Phi_{i}^{{\hat{\lambda }}_{e}⊙}\cdot \;\nabla {\left(\frac{1}{k_{\;B}T_{e}}\right)}^{⊙}-\Phi_{i}^{\Theta }\left(T_{e}-T_{h}\right), \end{array}$$

and the corresponding coefficients are solved by using tensorial integral equations. The transport coefficients are then typically obtained by using scalar products. The bracket operator defined by ${\left[\;\left[\xi_{h},\zeta_{h}\right]\;\right]}_{h}={\langle \;\langle f_{h}^{\left(0\right)}\xi_{h},I_{h}\zeta_{h}\rangle \;\rangle }_{h}$ is found to be hermitian ${\left[\;\left[\xi_{h},\zeta_{h}\right]\;\right]}_{h}={\bar{\left[\;\left[\zeta_{h},\xi_{h}\right]\;\right]}}_{h}$ and positive semi-definite $[\;\left[\xi_{h},\xi_{h}\right]\;]\geq 0$, and $[\;\left[\xi_{h},\xi_{h}\right]\;]=0$ if and only if $\xi_{h}$ is a collisional invariant.

From the multiscale asymptotic expansions, the equations governing the second-order corrector $\phi_{e}^{\left(2\right)}$ are finally found in the form

(14) $$\left\{\begin{array}{c} I_{e}\left(\phi_{e}^{\left(2\right)}\right)+\frac{q_{e}}{m_{e}}\left(C_{e}\wedge B\right)\cdot \;\partial_{C_{e}}\phi_{e}^{\left(2\right)}=\psi_{e}^{\left(2\right)},\\ {\langle \;\langle f_{e}^{\left(0\right)}\phi_{e}^{\left(2\right)},\psi^{l}\rangle \;\rangle }_{e}=0,\;l\in \left\{e,n+4\right\}. \end{array}\right.$$

After elimination of Burnett type terms, i.e., keeping only linear contributions, the right-hand side $\psi_{e}^{\left(2\right)}$ may be decomposed in the form

(15) $$\Psi_{e}^{\left(2\right)}=-\Psi_{e}^{\eta_{e}}\;:\;\nabla v_{h}-p_{e}\sum_{j\in H}\Psi_{e}^{D_{j}}\cdot \;d_{j}^{2}-{\tilde{\Psi }}_{e}^{\left(2\right)},$$

where the second-order diffusion driving force $d_{j}^{2}$ is given by $d_{j}^{2}=-V_{j}$ for j∈H, the scalar ${\tilde{\Psi }}_{e}^{\left(2\right)}$ is an isotropic function of $C_{\;e}$, and the expression of the coefficients $\Psi_{e}^{\eta_{e}}$, $\Psi_{e}^{D_{j}}$, and ${\tilde{\Psi }}_{e}^{\left(2\right)}$ may be found in the literature [22,23]. The perturbed distribution is then expanded in a similar way

(16) $$\phi_{e}^{\left(2\right)}=-\Phi_{e}^{\eta_{e}}\;:\;\nabla v_{h}-p_{e}\sum_{j\in H}\Phi_{e}^{D_{j}}\cdot \;d_{j}^{2}-{\tilde{\Phi }}_{e}^{\left(2\right)},$$

and the tensorial coefficients are solved by using integral equations with complex unknowns [22,23]. Since the perturbation $\phi_{e}^{\left(2\right)}$ only plays a role through the diffusion velocity $V_{e}^{1}=\int f_{e}^{\left(0\right)}\phi_{e}^{\left(2\right)}C_{\;e}dC_{\;e}$ and the associated conduction current, only the vector part of $\phi_{e}^{\left(2\right)}$ needs to be solved, proceeding as for the vector part of $\phi_{e}^{\left(1\right)}$.

### 2.4. First-Order Fluid Equations

Taking moments of the Boltzmann equation with the collisional invariants and using the gradient expansions of the perturbations $\phi_{e}^{\left(1\right)}$ and $\phi_{h}={\left(\phi_{i}^{\left(1\right)}\right)}_{i\in H}$ and of the vector part of $\phi_{e}^{\left(2\right)}$ yields the macroscopic equations at first order as well as the expression of the transport fluxes [22,23].

The electron conservation equations are found in the form

(17) $$\partial_{t}\rho_{e}+\nabla \cdot \;\left(\rho_{e}v_{h}+\rho_{e}V_{e}^{0}+\epsilon \rho_{e}V_{e}^{1}\right)=\epsilon m_{\epsilon }\omega_{\epsilon },$$

(18) $$\begin{array}{cc} \; & \partial_{t}E_{e}+\nabla \cdot \;\left(E_{e}v_{h}\right)=-p_{e}\nabla \cdot \;v_{h}-\nabla \cdot \;\left(Q_{e}^{0}+\epsilon \nabla \cdot \;Q_{e}^{1}\right)\\ \; & \;+\left(J_{e}^{0}+\epsilon J_{e}^{1}\right)\cdot \;E^{\prime }+\Delta E_{eh}^{0}+\epsilon \Delta E_{eh}^{1}, \end{array}$$

where $\rho_{e}=n_{e}m_{e}$ is the electron mass density and $E_{e}=\frac{3}{2}n_{e}k_{\;B}T_{e}$ the electron internal energy per unit volume. The electron diffusion velocities are given by $n_{e}V_{e}^{0}=\int C_{\;e}f_{e}^{\left(0\right)}\phi_{e}^{\left(1\right)}dc_{e}$ and $n_{e}V_{e}^{1}=\int C_{\;e}f_{e}^{\left(0\right)}\phi_{e}^{\left(2\right)}dc_{e}$, and the corresponding currents by $J_{e}^{0}=n_{e}q_{e}V_{e}^{0}$ and $J_{e}^{1}=n_{e}q_{e}V_{e}^{1}$. Similarly, the zeroth and first-order electron heat fluxes are given by $Q_{e}^{0}=\int \frac{1}{2}m_{e}{\left|C_{\;e}\right|}^{2}C_{\;e}f_{e}^{\left(0\right)}\phi_{e}^{\left(1\right)}dc_{e}$ and $Q_{e}^{1}=\int \frac{1}{2}m_{e}{\left|C_{\;e}\right|}^{2}C_{\;e}f_{e}^{\left(0\right)}\phi_{e}^{\left(2\right)}dc_{e}$ and the electron viscous tensor is found to be zero, $\Pi_{e}=0$. The molecular production rate of electrons by chemical reactions is given by $\omega_{\epsilon }=\int C_{e}dc_{e}$. The zeroth-order and first-order energy exchange terms may also be written in relaxation form [22,23].

The total electron diffusion velocity $V_{e}=V_{e}^{0}+\epsilon V_{e}^{1}$ is found in the form

(19) $$\begin{array}{c} V_{e}=-D_{ee}^{\|}d_{e}^{\|}-D_{ee}^{⊥}d_{e}^{⊥}-D_{ee}^{⊙}d_{e}^{⊙}-\theta_{e}^{\|}{\left(\nabla \log T_{e}\right)}^{\|}-\theta_{e}^{⊥}{\left(\nabla \log T_{e}\right)}^{⊥}\\ -\theta_{e}^{⊙}{\left(\nabla \log T_{e}\right)}^{⊙}-\sum_{i\in H}\epsilon \left(\alpha_{ie}^{\|}d_{i}^{2\|}+\alpha_{ie}^{⊥}d_{i}^{2⊥}+\alpha_{ie}^{⊙}d_{i}^{2⊙}\right), \end{array}$$

where $d_{e}=\left(\nabla p_{e}-n_{e}q_{e}E^{\prime }\right)/p_{e}$ and $d_{i}^{2}=-V_{i}$, i∈H. The last terms are second-order terms required for a O(ϵ) drift diffusion equation and have been termed the `Kolesnikov effect’ by Graille, Magin and Massot [22]. In the special situation $T_{h}=T_{e}$, these fluxes will be recovered from the kinetic theory of thermal plasmas in Section 4.

The heavy species governing equations are found in the form

(20) $$\begin{array}{cc} \; & \partial_{t}\rho_{i}+\nabla \cdot \;\left(\rho_{i}v_{h}+\epsilon \rho_{i}V_{i}\right)=\epsilon \;m_{i}\omega_{i},\;i\in H, \end{array}$$

(21) $$\begin{array}{cc} \; & \partial_{t}\left(\rho_{h}v_{h}\right)+\nabla \cdot \;\left(\rho_{h}v_{h}⊗v_{h}+pI\right)=-\epsilon \nabla \cdot \;\Pi_{h}+nqE^{\prime }+J\wedge B, \end{array}$$

(22) $$\begin{array}{cc} \; & \partial_{t}E_{h}+\nabla \cdot \;\left(E_{h}v_{h}\right)=-p_{h}\nabla \cdot \;v_{h}-\epsilon \nabla v_{h}\;:\;\Pi_{h}-\epsilon \nabla \cdot \;Q_{h}+\epsilon J_{h}\cdot \;E^{\prime } \end{array}$$

(23) $$\begin{array}{cc} \; & \;+\Delta E_{he}^{0}+\epsilon \Delta E_{he}^{1}. \end{array}$$

In these equations, $\rho_{h}=\sum_{i\in H}\rho_{i}$ is the heavy species mass density, $n=\sum_{i\in S}n_{i}$ the total number density, $nq=\sum_{i\in S}n_{i}q_{i}$ the total charge per unit volume, and $E_{h}$ the heavy species internal energy per unit volume. The diffusion velocities are given by $n_{i}V_{i}=\sum_{I\in Q_{i}}\int C_{\;i}f_{i}^{\left(0\right)}\phi_{i}^{\left(1\right)}dc_{i}$, i∈H, the diffusive current by $J_{h}=\sum_{i\in H}n_{i}q_{i}V_{i}$, and the total current by $J=J_{e}^{0}+\epsilon J_{e}^{1}+\epsilon J_{h}$. The heavy species viscous tensor is defined by $\Pi_{h}=\sum_{i\in H,I\in Q_{i}}\int m_{i}C_{\;i}⊗C_{\;i}f_{i}^{\left(0\right)}\phi_{i}^{\left(1\right)}dc_{i}$, and the heat flux by $Q_{h}=\sum_{i\in S,I\in Q_{i}}\int \left(\frac{1}{2}m_{i}{\left|C_{\;i}\right|}^{2}+E_{iI}\right)C_{\;i}f_{i}^{\left(0\right)}\phi_{i}^{\left(1\right)}dc_{i}$. The molecular production rate of the *i*th species by chemical reactions is given by $\omega_{i}=\sum_{I\in Q_{i}}\int C_{i}dc_{i}$. The first-order momentum conservation for electrons has also been used in the heavy species momentum conservation equations in order to eliminate the first-order average force exerted by the electrons on the heavy species [22,23]. Adding the electron and the heavy species conservation equations and letting $E=E_{e}+E_{h}$, $p=p_{e}+p_{h}$ and $Q=Q_{e}^{0}+\epsilon \nabla \cdot \;Q_{e}^{1}+\epsilon Q_{h}$, the total energy conservation equation is found in the form

(24) $$\begin{array}{c} \partial_{t}E+\nabla \cdot \;\left(Ev_{h}\right)=-p\nabla \cdot \;v_{h}-\epsilon \nabla v_{h}\;:\;\Pi_{h}-\nabla \cdot \;Q+J\cdot \;E^{\prime }, \end{array}$$

where we have used the natural relations $\Delta E_{he}^{0}+\Delta E_{eh}^{0}=0$ and $\Delta E_{he}^{1}+\Delta E_{eh}^{1}=0$. Substitution of the gradient expressions of the perturbed distributions then yields the expression of the transport fluxes as well as the expression of the transport coefficients.

The heavy species viscous tensor is obtained in the form

(25) $$\Pi_{h}=-\kappa_{h}\left(\nabla \cdot v_{h}\right)I-\eta_{h}\left(\nabla v_{h}+{\left(\nabla v_{h}\right)}^{t}-\frac{2}{3}\left(\nabla \cdot v_{h}\right)I\right),$$

where $\kappa_{h}$ denotes the volume viscosity and $\eta_{h}$ the shear viscosity. The diffusion velocities of the heavy species $V_{k}$, k∈H, are given by

(26) $$\begin{array}{cc} V_{i}= & -\sum_{j\in H}D_{ij}d_{j}-\theta_{i}\nabla \log T_{h}-D_{ie}^{\|}d_{e}^{\|}-D_{ie}^{⊥}d_{e}^{⊥}-D_{ie}^{⊙}d_{e}^{⊙}\\ \; & -\theta_{ie}^{\|}{\left(\nabla \log T_{e}\right)}^{\|}-\theta_{ie}^{⊥}{\left(\nabla \log T_{e}\right)}^{⊥}-\theta_{ie}^{⊙}{\left(\nabla \log T_{e}\right)}^{⊙}\;i\in H, \end{array}$$

where $d_{j}=\left(\nabla p_{j}-n_{j}q_{j}E^{\prime }\right)/p_{h}$ is the diffusion driving force of the *j*th species for j∈H. The diffusion velocities may also be written in the compact form $V_{i}=-\sum_{j\in H}D_{ij}d_{j}^{\prime }-\theta_{j}\nabla \log T_{h}$ where the modified diffusion driving force $d_{j}^{\prime }$ includes the polarized influence of the electrons $d_{j}^{\prime }=d_{j}-{\tilde{F}}_{je}/p_{h}$ with ${\tilde{F}}_{je}/p_{e}=-\alpha_{je}^{\|}d_{e}^{\|}-\alpha_{je}^{⊥}d_{e}^{⊥}-\alpha_{je}^{⊙}d_{e}^{⊙}-\chi_{je}^{\|}\nabla T_{e}^{\|}-\chi_{je}^{⊥}\nabla T_{e}^{⊥}-\chi_{je}^{⊙}\nabla T_{e}^{⊙}$. The polarization effects for the heavy species diffusion velocities thus only arise through the coupling with electrons. A similar expression is also found for the heavy species heat flux $Q_{h}$, and the entropy production associated with the fluid model may be shown to be non-negative [22,23].

### 2.5. Transport Linear Systems and Iterative Methods

The transport linear systems for the heavy species are similar to that of isotropic systems, since there is no magnetization for the heavy species [37]. These transport linear systems are obtained by using an orthogonal Galerkin solution of the linearized systems of Boltzmann equations for the perturbation $\phi_{h}^{\left(1\right)}$. The fast convergent iterative algorithms already developed for neutral mixtures may then be used in order to solve the transport linear systems associated with the heavy species.

In order to illustrate these algorithms, we only discuss the first approximation of the multicomponent diffusion coefficients of the heavy species for the sake of simplicity. The $n_{h}$ linear systems associated with first-order multicomponent diffusion are then of size $n_{h}$ and read

(27) $$\left\{\begin{array}{c} \Delta_{hh}\alpha_{h}^{{D\;}_{j}}=\beta_{h}^{{D\;}_{j}}\\ \langle \alpha_{h}^{{D\;}_{j}},y_{h}\rangle =0, \end{array}\right.\;j\in H,$$

and the diffusion coefficients are next given by $D_{ij}=\langle \alpha_{h}^{{D\;}_{i}},\beta_{h}^{{D\;}_{j}}\rangle =\alpha_{j}^{{D\;}_{i}}=\alpha_{i}^{{D\;}_{j}}$ for i,j∈H. The Stefan–Maxwell matrix $\Delta_{hh}$ has its coefficients given by

(28) $$\begin{array}{cc} \Delta_{ii}=\; & \sum_{j\in H,j\neq i}\frac{x_{i}x_{j}}{D_{ij}},\;i\in H, \end{array}$$

(29) $$\begin{array}{cc} \Delta_{ij}=\; & -\frac{x_{i}x_{j}}{D_{ij}},\;i,j\in H,\;i\neq j, \end{array}$$

where $x_{1},\ldots ,x_{n_{h}}$ denote the heavy species mole fractions, $D_{ij}$, i,j∈H, the heavy species binary diffusion coefficients, and $y_{h}={\left(y_{1},\ldots ,y_{n_{h}}\right)}^{t}$ the heavy species mass fraction vector [26,37]. The right-hand sides are $\beta_{h}^{{D\;}_{k}}=e_{h}^{k}-y_{h}$, k∈H, where $e_{h}^{k}$, k∈H, are the standard basis vectors of $R^{n_{h}}$. Denoting by N(A) and R(A) the nullspace and range of a matrix *A* and letting $u_{h}={\left(1,\ldots ,1\right)}^{t}\in R^{n_{h}}$, we have $N\left(\Delta_{hh}\right)=Ru_{h}$, $R\left(\Delta_{hh}\right)=u_{h}^{⊥}$, $N\left(D_{hh}\right)=Ry_{h}$, $R\left(D_{hh}\right)=y_{h}^{⊥}$ and $\langle y_{h},u_{h}\rangle =1$. Moreover, for any a>0, we also have $D_{hh}={\left(\Delta_{hh}+ay_{h}⊗y_{h}\right)}^{-1}\;-\;\left(1/a\right)u_{h}⊗u_{h}$ [38].

Letting $\Delta_{hh}=M_{h}-W_{h}$, where $M_{h}$ is diagonal and $M_{kk}=\Delta_{kk}/\left(1-y_{k}\right)$, $T_{h}=M_{h}^{-1}W_{h}$ and $P_{h}=Q_{h}^{t}=I_{h}-u_{h}⊗y_{h}$, we have $\Delta_{hh}D_{hh}=Q_{h}$ and the convergent asymptotic expansion

(30) $$D_{hh}\sum_{0\leq j<\infty }{\left(P_{h}T_{h}\right)}^{j}P_{h}M_{h}^{-1}P_{h}^{t}.$$

The first term $P_{h}M_{h}^{-1}P_{h}^{t}$ corresponds to a diagonal approximation $M_{h}^{-1}$ of the diffusion matrix with projectors $P_{h}$ and $P_{h}^{t}$ that guarantee mass conservation, and the two term expansion is more accurate [38]. Larger transport linear systems associated with higher order approximation of the multicomponent diffusion coefficients, with the thermal conductivity, with the thermal diffusion coefficients, or with more accurate electrical conductivities, are similarly investigated in the literature [28,29,38,39,40]. Libraries have also been developed for the evaluation of multitemperature transport coefficients, like the ${\mathrm{Mutation}}^{++}$ library [41].

## 3. Kinetic Theory of a One-Temperature Plasma

We again consider a multicomponent reactive magnetized plasma with polyatomic molecules, but the mass ratio between the species is now considered to remain of order unity. The corresponding kinetic theory then involves a single collision time and the characteristic quantities of all species are identical. It is also naturally assumed that the species share the same temperature, so that the plasma is a thermal plasma.

Important differences with the previous regime are notably that the thermal velocities of all the species are of order unity—including the electrons, that the plasma is a thermal plasma, that there is a single energy collisional invariant, and that the magnetic force terms $\left(c_{k}-v\right)\wedge B\cdot \nabla_{\;\;c_{k}}f_{k}$ must now be taken into account at the zeroth order for all the species. As a consequence, all the species have nonisotropic transport properties, the viscous tensor involves all second-order tensors that can be built from the rate of strain matrix and the magnetic field vector, and there are five shear viscosities.

A generalized Chapman–Enskog procedure is used and yields the macroscopic equations and the transport fluxes, as well as the source terms [28,29]. The equilibrium distributions are again Maxwellian distributions, the reference velocity is the usual mass average velocity v, and the transport fluxes of all the species are found to be nonisotropic.

### 3.1. Rescaled Boltzmann Equations

The notation of the previous section is used so that $f_{i}\left(t,x,c_{i},i\right)$ denotes the distribution function of the *i*th species, *t* the time, x the three-dimensional spatial coordinate, $c_{i}$ the velocity of the particles of the *i*th species, i the index for the internal energy state of the *i*th species, S={1,…,n} the species indexing set, and *n* the number of species, and the characteristic quantities are written with the ^★^ superscript. The characteristic quantities are the mass $m^{★}$, the temperature $T^{★}$, the number density $n^{★}$, the fluid velocity $v^{★}$, the elastic or inelastic cross sections $\sigma^{★}$, and the free path $l^{★}=1/n^{★}\sigma^{★}$. The thermal velocities are of order $C^{★}={\left(k_{\;B}T^{★}/m^{★}\right)}^{1/2}$ and there are only two characteristic times, one for the particle dynamics $t^{★}/\epsilon$, and one for the fluid dynamics $t^{★}$. The Mach number is assumed to be of order unity with $v^{★}=C^{★}$. The corresponding fluid length scale is $L^{★}=v^{★}t^{★}$ and the Knudsen number is given by $\mathrm{Kn}=l^{★}/L^{★}=\epsilon$. The electric field is such that the reference electrical and thermal energies are of the same order of magnitude $q^{★}E^{★}L^{★}=k_{\;B}T^{★}$, the magnetic field is estimated with the Hall numbers of particles $\beta_{i}=q^{★}B^{★}t^{★}/m^{★}=1$, i∈S, and the chemistry terms are assumed to be of order O(ϵ).

The corresponding rescaled Boltzmann equations are then found in the form

(31) $$\partial_{t}f_{i}+c_{i}\cdot \nabla f_{i}+\frac{q_{i}}{m_{i}}E^{\prime }\cdot \nabla_{\;\;c_{i}}f_{i}+\frac{1}{\epsilon }\frac{q_{i}}{m_{i}}\left(C_{i}\wedge B\right)\cdot \nabla_{\;\;c_{i}}f_{i}=\frac{1}{\epsilon }\sum_{j\in S}J_{ij}+\epsilon \;C_{i},\;k\in S,$$

where $q_{i}$ denotes the charge of the particles of the *i*th species—that is zero for neutral species, $m_{i}$ the mass of the particles of the *i*th species, v the mass average fluid velocity, $E^{\prime }=E+v\wedge B$ the electric field in the fluid reference frame, E the electric field, B the magnetic field, $C_{i}=c_{i}-v$ the thermal speed of the particles of the *i*th species, $J_{ij}$, i,j∈S, the scattering collision operators and $C_{i}$, i∈S, the reactive collision operators. These collision operators may involve all particles and are detailed in the literature, and the details are omitted [3,28,29,37]. The electron dynamics is now not faster than the heavier species in comparison with the rescaled Boltzmann Equations (1) and (2).

### 3.2. Chapman–Enskog Expansion

For two families of tensors $\xi ={\left(\xi_{i}\right)}_{i\in S}$ and $\zeta ={\left(\zeta_{i}\right)}_{i\in S}$, where $\xi_{i}$ and $\zeta_{i}$ depend on $C_{\;i}$ and i, we define the scalar product by

$$\langle \;\langle \xi ,\zeta \rangle \;\rangle =\sum_{i\in S}\sum_{I\in Q_{i}}\int \xi_{i}⊙{\bar{\zeta }}_{i}\;dc_{i},$$

where $\xi_{i}⊙{\bar{\zeta }}_{i}$ is the contracted product between the tensor $\xi_{i}$ and the complex conjugate tensor ${\bar{\zeta }}_{i}$. The scalar product is defined for families of complex tensors, as such quantities naturally arise in the solution of Boltzmann linearized equations in the presence of magnetic fields. The collision invariants are given by $\psi^{l}={\left(\delta_{li}\right)}_{i\in S}$ for l∈S, $\psi^{n+\nu }={\left(m_{i}C_{i\nu }\right)}_{i\in S}$ for ν=1,2,3, and $\psi^{n+4}={\left(\frac{1}{2}m_{i}{\left|C_{\;i}\right|}^{2}+E_{iI}\right)}_{i\in S}$.

An approximate solution to the Boltzmann equations is obtained by using the Enskog expansion

(32) $$f_{i}=f_{i}^{\left(0\right)}\left(1+\epsilon \phi_{i}^{\left(1\right)}+O\left(\epsilon^{2}\right)\right),\;i\in S,$$

with the Enskog constraints $\langle \;\langle f^{\left(0\right)},\psi^{l}\rangle \;\rangle =\langle \;\langle f,\psi^{l}\rangle \;\rangle$ for l∈{1,…,n+4}.

The zeroth-order species distribution functions $f^{\left(0\right)}={\left(f_{i}^{\left(0\right)}\right)}_{i\in S}$ are found to be Maxwellian distributions [26,28,29]

(33) $$f_{i}^{\left(0\right)}=n_{i}{\left(\frac{m_{i}}{2\pi k_{\;B}T}\right)}^{\frac{3}{2}}\frac{a_{iI}}{Z_{i}^{\mathrm{int}}}\exp\left(-\frac{m_{i}}{2k_{\;B}T}{\left|c_{i}-v\right|}^{2}-\frac{E_{iI}}{k_{\;B}T}\right),\;i\in S,$$

where $n_{i}$ is the number density of the *i*th species, $m_{i}$ the mass of the particles of the *i*th species, $k_{\;B}$ the Boltzmann constant, $c_{i}$ the velocity of the particles of the *i*th species, v the fluid velocity, *T* the temperature, $E_{iI}$ the energy of the *i*th species in the ith state, $a_{iI}$ the degeneracy of the ith state, and $Z_{i}^{\mathrm{int}}=\sum_{I\in Q_{i}}a_{iI}\exp\left(-\frac{E_{iI}}{k_{\;B}T}\right)$ the internal energy partition function of the *i*th species. The zeroth-order equations are found to be the hyperbolic Euler equations [28,29].

The thermodynamic properties may be evaluated from the Maxwellian distributions, and it is found that $p=nk_{\;B}T$, $E=\sum_{i\in S}n_{i}{\bar{E}}_{i}$ where ${\bar{E}}_{i}=\frac{3}{2}k_{\;B}T+{\bar{E}}_{i}^{\mathrm{int}}$ and ${\bar{E}}_{i}^{\mathrm{int}}=\sum_{I\in Q_{i}}\frac{a_{iI}E_{iI}}{Z_{i}^{\mathrm{int}}}\exp\left(-\frac{E_{iI}}{k_{\;B}T}\right)$. Further introducing the translational energy partition function of the *i*th species per unit volume $Z_{i}^{\mathrm{tr}}={\left(2\pi m_{i}k_{\;B}T/{h_{P}}^{2}\right)}^{3/2}$, the complete partition function is $Z_{i}=Z_{i}^{\mathrm{tr}}Z_{i}^{\mathrm{int}}$ for i∈S. The Gibbs function per unit volume of the *i*th species is then $G_{i}=k_{\;B}T\log\left(n_{i}/Z_{i}\right)$ and the corresponding entropy per unit volume reads $S_{i}=\frac{5}{2}k_{\;B}+\frac{{\bar{E}}_{i}}{T}-k_{\;B}\log\left(n_{i}/Z_{i}\right)$ for i∈S.

### 3.3. Linearized Kinetic Equations

Application of the Chapman–Enskog method then leads at first order to the linearized Boltzmann equations governing the species perturbed distribution functions $\phi^{\left(1\right)}={\left(\phi_{i}^{\left(1\right)}\right)}_{i\in S}$. Denoting by $I={\left(I_{i}\right)}_{i\in S}$ the linearized collision operator, the linearized equations governing $\phi^{\left(1\right)}$ are in the form

(34) $$\left\{\begin{array}{c} I_{i}\left(\phi^{\left(1\right)}\right)+\frac{q_{i}}{m_{i}}\left(C_{i}\wedge B\right)\cdot \;\partial_{c_{i}}\phi_{i}^{\left(1\right)}+\sum_{\begin{array}{c} j\in S\\ j\in Q_{j} \end{array}}\;\;\;\frac{m_{i}C_{\;i}q_{j}}{\rho k_{\;B}T}\cdot \;\;\;\int f_{j}^{\left(0\right)}\phi_{j}^{\left(1\right)}C_{\;j}\wedge Bdc_{j}=\psi_{i},\;i\in S,\\ \langle \;\langle f^{\left(0\right)}\phi^{\left(1\right)},\psi^{l}\rangle \;\rangle =0,\;1\leq l\leq n+4. \end{array}\right.$$

The heavy species kinetic equations now contain magnetic terms in comparison with the two-temperature kinetic model (12). An important property is then the isotropy of the linearized Boltzmann operator $I={\left(I_{i}\right)}_{i\in S}$ that converts a tensor constructed from ${\left(C_{i}\right)}_{i\in S}$ into another tensor of the same type [26]. The bracket operator is defined by $\left[\;\left[\xi ,\zeta \right]\;\right]=\langle \;\langle f^{\left(0\right)}\xi ,I\left(\zeta \right)\rangle \;\rangle$, where $\xi ={\left(\xi_{i}\right)}_{i\in S}$, $\zeta ={\left(\zeta_{i}\right)}_{i\in S}$, and $\xi_{i}$ and $\zeta_{i}$ depend on $C_{\;i}$ and i. This bracket operator is hermitian $\left[\;\left[\xi ,\zeta \right]\;\right]=\bar{\left[\;[\zeta ,\xi ]\;\right]}$ and positive semi-definite [[ξ,ξ]]≥0, and its kernel is spanned by the collisional invariants, that is, [[ξ,ξ]]=0 implies that ξ is a tensorial collisional invariant, so that all its tensorial components are scalar collisional invariants.

The right-hand side $\psi^{\left(1\right)}={\left(\psi_{i}^{\left(1\right)}\right)}_{i\in S}$ is obtained in the form

$$\Psi_{i}^{\left(1\right)}=-\Psi_{i}^{\eta }\;:\;\nabla v-\frac{1}{3}\Psi_{i}^{\kappa }\nabla \cdot \;v-\sum_{j\in S}\Psi_{i}^{D_{j}}\cdot \;\left(\nabla p_{i}-n_{i}q_{i}E^{\prime }\right)-\Psi_{i}^{\hat{\lambda }}\cdot \;\nabla \left(\frac{1}{k_{\;B}T}\right),$$

where $p_{i}$ denotes the partial pressure of the *i*th species and the functions $\Psi_{i}^{\eta }$, $\Psi_{i}^{\kappa }$, $\Psi_{i}^{D_{j}}$, and $\Psi_{i}^{\hat{\lambda }}$ may be found in the literature [28,29,37]. By linearity and isotropy of the linearized Boltzmann operator $I={\left(I_{i}\right)}_{i\in S}$, the solution $\phi ={\left(\phi_{i}\right)}_{i\in S}$ is expanded in the form

(35) $$\phi_{i}=-\phi_{i}^{\eta }\;:\;\nabla v-\frac{1}{3}\phi_{i}^{\kappa }\nabla \cdot \;v-\sum_{j\in S}\phi_{i}^{D_{j}}\cdot \;\left(\nabla p_{j}-n_{j}q_{j}E^{\prime }\right)-\phi_{i}^{\hat{\lambda }}\cdot \;\nabla \left(\frac{1}{k_{\;B}T}\right),$$

and the coefficients $\phi^{\eta }={\left(\phi_{i}^{\eta }\right)}_{i\in S}$, $\phi^{\kappa }={\left(\phi_{i}^{\kappa }\right)}_{i\in S}$, $\phi^{D_{j}}={\left(\phi_{i}^{D_{j}}\right)}_{i\in S}$, j∈S, $\phi_{i}^{\hat{\lambda }}={\left(\phi_{i}^{\hat{\lambda }}\right)}_{i\in S}$ then satisfy tensorial integral equations. The resulting integro-differential equations for the coefficients may then be transformed into integral equations by using the tensorial structure in terms of the thermal velocities ${\left(C_{\;i}\right)}_{i\in S}$ [26,28,29].

The solution $\phi^{\eta }={\left(\phi_{i}^{\eta }\right)}_{i\in S}$ with μ=η is such that $\phi_{i}^{\eta }$ is composed from all symmetric traceless second-order tensors created from the vector $C_{i}=\sqrt{m_{i}/2k_{\;B}T}\;C_{\;i}$ and the pseudo-vector B which form a space of dimension five. A new expansion of $\phi^{\eta }$ using five tensors has been introduced and has clarified the solution of the corresponding tranport linear system using traditionally six linearly dependent tensors [28,29].

For illustration, we investigate more closely the kinetic equations associated with the diffusion velocities in order to present the analysis of magnetized-type integral equations. The vector perturbed distribution functions $\phi^{D_{j}}={\left(\phi_{i}^{D_{j}}\right)}_{i\in S}$ are expanded in the form

(36) $$\phi_{i}^{D_{j}}=\phi_{i}^{D_{j}\left(1\right)}C_{\;i}+\phi_{i}^{D_{j}\left(2\right)}C_{\;i}\wedge B+\phi_{i}^{D_{j}\left(3\right)}C_{\;i}\cdot \;B\;B.$$

Then, using the tensorial structure (36), the differential–integral Equation (34) are transformed into integral equations [26,28,29]. These integral equations are then reformulated by using complex numbers that conveniently represent the rotation associated with the magnetic field. As a result, defining $\varphi_{i}^{D_{j}\left(1\right)}=\phi_{i}^{D_{j}\left(1\right)}+B^{2}\phi_{i}^{D_{j}\left(3\right)}$ and $\varphi_{i}^{D_{j}\left(2\right)}=\phi_{i}^{D_{j}\left(1\right)}+iB\phi_{i}^{D_{j}\left(2\right)}$ as well as $\varphi^{D_{j}\left(1\right)}={\left(\varphi_{i}^{D_{j}\left(1\right)}C_{\;i}\right)}_{i\in S}$ and $\varphi^{D_{j}\left(2\right)}={\left(\varphi_{i}^{D_{j}\left(2\right)}C_{\;i}\right)}_{i\in S}$, the integral equation for $\varphi^{D_{j}\left(1\right)}$ reads for any j∈S

(37) $$\left\{\begin{array}{c} I\left(\varphi^{D_{j}\left(1\right)}\right)=\Psi^{D_{j}},\\ \langle \;\langle f^{\left(0\right)}\varphi^{D_{j}\left(1\right)},\psi^{l}\rangle \;\rangle =0,\;l\in \left\{1,\ldots ,n+4\right\}, \end{array}\right.$$

and the complex integral equation for $\varphi^{D_{j}\left(2\right)}$ reads for all j∈S

(38) $$\left\{\begin{array}{c} \left(I+iBI^{B}\right)\left(\varphi^{D_{j}\left(2\right)}\right)=\Psi^{D_{j}},\\ \langle \;\langle f^{\left(0\right)}\varphi^{D_{j}\left(2\right)},\psi^{l}\rangle \;\rangle =0,\;l\in \left\{1,\ldots ,n+4\right\}, \end{array}\right.$$

where the operator $I^{B}={\left(I_{i}^{B}\right)}_{i\in S}$ is given for $u={\left(u_{i}\right)}_{i\in S}$ by

(39) $$I_{i}^{B}\left(u\right)=\frac{q_{i}}{m_{i}}u_{i}-\sum_{\begin{array}{c} j\in S\\ j\in Q_{j} \end{array}}\frac{m_{i}C_{\;i}q_{j}}{3\rho k_{\;B}T}\int f_{j}^{\left(0\right)}u_{j}\cdot \;C_{\;j}\;dc_{j},\;i\in S.$$

We may also rewrite (36) in the form

(40) $$\phi_{i}^{D_{j}}=\left(\varphi_{i}^{D_{j}\left(1\right)}\;B⊗B+ℜ\left(\varphi_{i}^{D_{j}\left(2\right)}\right)\;\left(I-B⊗B\right)-ℑ\left(\varphi_{i}^{D_{j}\left(2\right)}\right)\;R\left(B\right)\right)C_{\;i},$$

or, equivalently, $\phi_{i}^{D_{j}}=ℜ\left(\varphi_{i}^{D_{j}\left(1\right)}\;C_{\;i}^{\|}+\varphi_{i}^{D_{j}\left(2\right)}\;\left(C_{\;i}^{⊥}+iC_{\;i}^{⊙}\right)\right)$, where for any complex number *z*, the symbols ℜ(z) and ℑ(z) denote, respectively, the real and imaginary parts. The vector unknown $\phi^{\hat{\lambda }}$ is also expanded in a similar way, and substitution of the expansions of $\phi^{\hat{\lambda }}$ and $\phi^{D_{j}}$, j∈S in the definition of the multicomponent fluxes finally yield the species diffusion velocities as well as the heat flux [28,29].

### 3.4. First-Order Fluid Equations

The macroscopic conservation equations at first order are obtained by taking the scalar product of Boltzmann equations with collisional invariants and by keeping the zeroth and first-order terms.

The first-order governing equations are obtained in the form

(41) $$\begin{array}{cc} \; & \partial_{t}\rho_{i}+\nabla \cdot \;\left(\rho_{i}v+\epsilon \rho_{i}V_{i}\right)=\epsilon m_{i}\omega_{i},\;i\in S, \end{array}$$

(42) $$\begin{array}{cc} \; & \partial_{t}\left(\rho v\right)+\nabla \cdot \;\left(\rho v⊗v+pI\right)=-\epsilon \nabla \cdot \;\Pi +nqE^{\prime }+\epsilon J\wedge B, \end{array}$$

(43) $$\begin{array}{cc} \; & \partial_{t}E+\nabla \cdot \;\left(Ev\right)=-p\nabla \cdot \;v-\epsilon \nabla v\;:\;\Pi -\epsilon \nabla \cdot \;Q+\epsilon J\cdot \;E^{\prime }, \end{array}$$

where $\rho_{i}$ denotes the partial density of the *i*th species, v the mixture fluid velocity, $V_{i}$ the diffusion velocity of the *i*th species, $m_{i}$ the mass of the *i*th species, $\omega_{i}$ the molecular production of the *i*th species, $\rho =\sum_{i\in S}\rho_{i}$ the total mass density, p the pressure, Π the viscous tensor, nq the charge per unit volume, $E^{\prime }=E+v\wedge B$ the electric field in the fluid reference frame, E the electric field, B the magnetic field, E the energy per unit volume, and J the conduction courant. The diffusion velocity of the *i*th species is defined by $n_{i}V_{i}=\sum_{I\in Q_{i}}\int C_{\;i}f_{i}^{\left(0\right)}\phi_{i}^{\left(1\right)}\;dc_{i}$, the chemical source term of the *i*th species by $\omega_{i}=\sum_{I\in Q_{i}}\int C_{i}\left(f^{\left(0\right)}\right)dc_{i}$, the total charge per unit volume by $q=\sum_{i\in S}n_{i}q_{i}$, the conduction current by $J=\sum_{i\in S}n_{i}q_{i}V_{i}$, the viscous tensor by $\Pi =\sum_{i\in S,I\in Q_{i}}\int m_{i}C_{\;i}⊗C_{\;i}f_{i}^{\left(0\right)}\phi_{i}^{\left(1\right)}\;dc_{i}$ and the heat flux vector by $Q=\sum_{i\in S,I\in Q_{i}}\int \left(\frac{1}{2}m_{i}{\left|C_{\;i}\right|}^{2}+E_{iI}\right)C_{\;i}f_{i}^{\left(0\right)}\phi_{i}^{\left(1\right)}\;dc_{i}$.

The viscous tensor is found in the form

(44) $$\begin{array}{cc} \Pi = & -\kappa \nabla \cdot \;v\;I-\eta_{1}S-\eta_{2}\left(R(B)\;S-S\;R(B)\right)-\eta_{3}\left(-R(B)\;S\;R(B)+\langle SB,B\rangle \;B⊗B\right)\\ & -\eta_{4}\left(S\;B⊗B+B⊗B\;S-2\langle SB,B\rangle \;B⊗B\right)-\eta_{5}\left(B⊗B\;S\;R(B)-R(B)\;S\;B⊗B\right), \end{array}$$

where $S=\left(\nabla v+\nabla v^{t}\right)-\frac{2}{3}\left(\nabla \cdot \;v\right)\;I$ is the strain rate tensor, B=B/|B|, R(B) the rotation matrix such that R(B)x=B∧x, κ the volume viscosity and $\eta_{1},\ldots ,\eta_{5}$ the five shear viscosities.

Substituting the expansion (35) into the identity $V_{i}=k_{\;B}T\langle \;\langle \Psi^{D_{i}},f^{\left(0\right)}\phi^{\left(1\right)}\rangle \;\rangle$, only the vector terms $\phi^{D_{j}}$ for j∈S and $\phi^{\hat{\lambda }}$ yield non-null contributions. Expanding $\phi^{D_{j}}$, j∈S, and $\phi^{\hat{\lambda }}$, using isotropy and denoting by $d_{j}=\left(\nabla p_{j}-n_{j}q_{j}E^{\prime }\right)/p$ the unconstrained diffusion driving force of the *j*th species, the species diffusion velocities are obtained in the form

(45) $$\begin{array}{ccc} V_{i} & = & -\sum_{j\in S}\left(D_{ij}^{\|}d_{j}^{\|}+D_{ij}^{⊥}d_{j}^{⊥}+D_{ij}^{⊙}d_{j}^{⊙}\right)\\ & & \;-\left(\theta_{i}^{\|}{\left(\nabla \log T\right)}^{\|}+\theta_{i}^{⊥}{\left(\nabla \log T\right)}^{⊥}+\theta_{i}^{⊙}{\left(\nabla \log T\right)}^{⊙}\right), \end{array}$$

where for any vector x, we denote by $x^{\|}$, $x^{⊥}$, and $x^{⊙}$ the associated vectors $x^{\|}=x\cdot \;B\;\;B$, $x^{⊥}=x-x^{\|}$ and $x^{⊙}=B\wedge x$. Similar results may also be obtained for the heat flux of the mixture [28,29]. The transport coefficients may typically be expressed in terms of the perturbation $\varphi^{D_{j}\left(1\right)}$ and $\varphi^{D_{j}\left(2\right)}$ with

$$D_{ij}^{\|}=\frac{pk_{\;B}T}{3}\langle \;\langle f^{\left(0\right)}\varphi^{D_{j}\left(1\right)},\Psi^{D_{i}}\rangle \;\rangle ,\;D_{ij}^{⊥}+iD_{ij}^{⊙}=\frac{pk_{\;B}T}{3}\langle \;\langle f^{\left(0\right)}\varphi^{D_{j}\left(2\right)},\Psi^{D_{i}}\rangle \;\rangle .$$

These multicomponent diffusion coefficients parallel $D_{ij}^{\|}$, perpendicular $D_{ij}^{⊥}$, and transverse $D_{ij}^{⊙}$ to the magnetic field are symmetric, and the entropy production associated with all dissipative effects has been shown to be non-negative [28,29].

The one-temperature diffusion velocity (45) is finally found to be similar to that of electrons (19) from the two-temperature theory with the second-order correctors represented by the cross diffusion terms. The one-temperature diffusion velocity (45) still differs from that of heavy species from the two-temperature theory (26), where magnetization has been lost due to the scaling except for the coupling terms with electrons.

### 3.5. Example of a Magnetized Transport Linear System

In order to illustrate the evaluation of transport coefficients in magnetized systems, we consider again the case of multicomponent diffusion coefficients associated with the diffusion velocities (45). Our aim is to present a typical example of a magnetized transport linear system and, for the sake of simplicity, only the first approximation of the species diffusion velocities obtained with a variational approximation space of dimension *n* is considered. The analysis of more complex transport linear systems associated with diffusion coefficients, thermal diffusion coefficients or thermal conductivities and involving transport linear systems of dimension $2n+n^{p}$, where $n^{p}$ is the number of polyatomic species, is entirely similar, as well as that of transport linear systems associated with the evaluation of the five shear viscosities or with the volume viscosity [28,29,38,39,40].

The simplest variational approximation space to be considered for the diffusion coefficients is the space spanned by $\phi^{1000k}$, k∈S, where $\phi^{1000k}={\left(\delta_{ik}\sqrt{m_{i}/2k_{\;B}T}\;C_{\;i}\right)}_{i\in S}$. The vector integral equations associated with multicomponent diffusion coefficients (37) and (38) are in the form $I\left(\varphi^{D_{j}\left(1\right)}\right)=\Psi^{D_{j}}$ and $\left(I+iBI^{B}\right)\left(\varphi^{D_{j}(2}\right)=\Psi^{D_{j}}$ for j∈S, where $\Psi^{D_{j}}$ may be decomposed in the form

$$\Psi^{D_{j}}=\sum_{k\in S}\sqrt{2/m_{k}k_{\;B}T}\left(\delta_{jk}-y_{k}\right)/n_{k}\phi^{1000k},\;j\in S.$$

For convenience, $\varphi^{D_{j}\left(l\right)}$ is taken in the form

$$\varphi^{D_{j}\left(l\right)}=\frac{\sqrt{2}}{p\sqrt{k_{\;B}T}}\sum_{k\in S}\sqrt{m_{k}}\alpha_{k}^{D_{j}\left(l\right)}\phi^{1000k},\;l\in \left\{1,2\right\},\;j\in S.$$

The matrix associated with the variational procedure is denoted by Δ and is rescaled such that

$$\Delta_{kl}=(2\sqrt{m_{k}m_{l}}/3p\left[\;\left[\phi^{1000k},\phi^{1000l}\right]\;\right],\;k,l\in S,$$

with a similar rescaling for $\Delta^{B}$. We also rescale the right member

$$\beta_{k}^{D_{j}}=\left(\sqrt{2m_{k}k_{\;B}T}/3\right)\langle \;\langle f^{\left(0\right)}\phi^{1000k},\Psi^{D_{j}}\rangle \;\rangle ,\;k\in S.$$

We then have $\Delta ,\Delta^{B}\in R^{n,n}$, $\beta^{D_{j}},\alpha^{D_{j}\left(1\right)}\in R^{n}$, $\alpha^{D_{j}\left(2\right)}\in C^{n}$, and the linear systems for $\alpha^{D_{j}\left(1\right)}={\left(\alpha_{k}^{D_{j}\left(1\right)}\right)}^{k\in S}$ and $\alpha^{D_{j}\left(2\right)}={\left(\alpha_{k}^{D_{j}\left(2\right)}\right)}_{k\in S}$ are in the form

(46) $$\left\{\begin{array}{cc} \Delta \;\alpha^{D_{j}\left(1\right)} & =\beta^{D_{j}},\\ \langle \alpha^{D_{j}\left(1\right)},y\rangle & =0, \end{array}\right.\;\left\{\begin{array}{cc} \left(\Delta +i\Delta^{B}\right)\;\alpha^{D_{j}\left(2\right)} & =\beta^{D_{j}},\\ \langle \alpha^{D_{j}\left(2\right)},y\rangle & =0. \end{array}\right.$$

We have denoted by $y={\left(y_{1},\ldots ,y_{n}\right)}^{t}$ the mass fraction vector with 〈y,u〉=1, where $u={\left(1,\ldots ,1\right)}^{t}$, and by $\langle x,y\rangle =\sum_{k\in S}x_{k}{\bar{y}}_{k}$ the Hermitian scalar product. The coefficients of the matrix Δ are found to be

$$\Delta_{kk}=\sum_{\begin{array}{c} l\in S\\ l\neq k \end{array}}\frac{x_{k}x_{l}}{D_{kl}},\;k\in S,\;\;\Delta_{kl}=-\frac{x_{k}x_{l}}{D_{kl}},\;k,l\in S,\;\;k\neq l,$$

where $D_{kl}$ is the binary diffusion coefficient of the species pair (k,l), and $x_{i}$ is the mole fraction of the *i*th species with $y_{i}=m_{i}x_{i}/\sum_{j\in S}m_{j}x_{j}$. The matrix $\Delta^{B}$ is found to be

$$\Delta^{B}=\left(I-y⊗u\right)\;D^{B}\left(I-u⊗y\right),$$

where $D^{B}$ is the diagonal matrix $D_{kl}^{B}=\delta_{kl}n_{k}q_{k}B/p$ and the right members $\beta^{D_{j}}={\left(\beta_{k}^{D_{j}}\right)}_{k\in S}$ are given by

$$\beta_{k}^{D_{j}}=\delta_{jk}-y_{k},\;k,j\in S.$$

The real matrix Δ is symmetric positive definite, N(Δ)=Ru, $R\left(\Delta \right)=u^{⊥}$ in $R^{n}$, whereas the complex matrix $\Delta +i\Delta^{B}$ is such that $N(\Delta +i\Delta^{B})=Cu$, $R\left(\Delta +i\Delta^{B}\right)=u^{⊥}$ in $C^{n}$, where the nullspace and range of a matrix *A* are denoted by N(A) and R(A), respectively [28,29,37,38,39,40]. The resulting matrices $D^{\|}$, $D^{⊥}$, $D^{⊙}$, are given by

(47) $$\begin{array}{cc} D_{ij}^{\|}\; & =\langle \alpha^{D_{j}\left(1\right)},\beta^{D_{i}}\rangle =\alpha_{i}^{D_{j}\left(1\right)}=\alpha_{j}^{D_{i}\left(1\right)}, \end{array}$$

(48) $$\begin{array}{cc} D_{ij}^{⊥}+iD_{ij}^{⊙}\; & =\langle \alpha^{D_{j}\left(2\right)},\beta^{D_{i}}\rangle =\alpha_{i}^{D_{j}\left(2\right)}=\alpha_{j}^{D_{i}\left(2\right)}. \end{array}$$

In addition, for all a>0, we have $D^{\|}={\left(\Delta +ay⊗y\right)}^{-1}\;-\;\left(1/a\right)u⊗u$ as well as $D^{⊥}+iD^{⊙}={\left(\Delta +i\Delta^{B}+ay⊗y\right)}^{-1}\;-\;\left(1/a\right)u⊗u$.

### 3.6. Iterative Methods

In order to address the use of iterative techniques for solving the transport linear systems, either isotropic or magnetized, we specifically consider the linear systems of size *n* associated with diffusion velocities. We refer to the literature for other transport linear systems as well as numerical simulations for reentry or combustion applications [28,29,37,38,39,40]. We first discuss an acceleration technique for the linear systems associated with transport coefficients parallel to the magnetic field $D^{\|}$ that are similar to that of standard isotropic systems. We next discuss the linear systems associated with transport coefficients perpendicular or transverse to the magnetic field $D^{⊥}+iD^{⊙}$ that involve complex linear systems.

In order to solve the system (46) involving the matrix Δ, we introduce a splitting Δ=M−W, where *M* is diagonal and $M_{kk}=\Delta_{kk}/\left(1-y_{k}\right)$, $T=M^{-1}W$ and $P=Q^{t}=I-u⊗y$. We then have ΔD=Q and the convergent asymptotic expansion

(49) $$D^{\|}=\sum_{0\leq j<\infty }{\left(PT\right)}^{j}PM^{-1}P^{t}.$$

The projector *P* is needed in the expansion, since the spectral radius of PT is strictly lower than unity, whereas that of the iteration matrix T is unity [38]. The two terms’ expansion is especially interesting since it yields $n^{2}$ coefficients within $O\left(n^{2}\right)$ operations [28,29,37,38,39,40]. However, the good convergence rates observed for neutral species mixtures deteriorate when ionization levels increase [39,42].

In order to improve the convergence of the iterates, we let $\Delta_{1}=\Delta$, $D_{1}=D^{\|}$, $z_{1}=y$, $u_{1}=u$, and $Q_{1}=I_{n}-z_{1}⊗u_{1}$, so that $\langle z_{1},u_{1}\rangle =1$, $N\left(\Delta_{1}\right)=Ru_{1}$, $N\left(D_{1}\right)=Rz_{1}$, and $\Delta_{1}D_{1}=Q_{1}$. Letting I be the set of ionized species, we assume naturally that there are neutral as well as ionized species in the mixture so that I is not empty and differs from S. We then consider the vector $u_{2}^{*}$ with components ${\left(u_{2}^{*}\right)}_{i}=1$ if i∈I, and ${\left(u_{2}^{*}\right)}_{i}=0$ if i∉I, and define $u_{2}=u_{2}^{*}-\frac{\langle u_{2}^{*},z_{1}\rangle }{\langle z_{1},u_{1}\rangle }u_{1}$ in such a way that $\langle u_{2},z_{1}\rangle =0$ and $u_{2}\notin Ru_{1}$ since $u_{2}^{*}\notin Ru_{1}$. It is then evaluated that $\langle u_{2}^{*},z\rangle =y_{c}=\sum_{k\in I}y_{k}$ so that $u_{2}$ is given by ${\left(u_{2}\right)}_{k}=1-y_{c}$ if k∈I and ${\left(u_{2}\right)}_{k}=-y_{c}$ otherwise. Letting $z_{2}=\Delta u_{2}$, we then have by construction $\langle u_{1},z_{2}\rangle =0$ and $\langle z_{2},u_{2}\rangle =\langle \Delta u_{2},u_{2}\rangle >0$ since $u_{2}\notin Ru_{1}$. We may then define

$$\Delta_{2}=\Delta_{1}-\frac{z_{2}⊗z_{2}}{\langle u_{2},z_{2}\rangle },\;D_{2}=D_{1}-\frac{u_{2}⊗u_{2}}{\langle u_{2},z_{2}\rangle },\;Q_{2}=Q_{1}-\frac{u_{2}⊗z_{2}}{\langle u_{2},z_{2}\rangle }.$$

It is then established after some algebra that $\Delta_{2}$ and $D_{2}$ are positive semi-definite, that $\Delta_{2}D_{2}=Q_{2}$ and $D_{2}\Delta_{2}=P_{2}=Q_{2}^{t}$, and that the nullspaces of $\Delta_{2}$, $D_{2}$ and $Q_{2}$ are now two-dimensional with $N\left(\Delta_{2}\right)=\mathrm{span}\left\{u_{1},u_{2}\right\}$, $N\left(D_{2}\right)=\mathrm{span}\left\{z_{1},z_{2}\right\}$, and $N\left(Q_{2}\right)=\mathrm{span}\left\{u_{1},u_{2}\right\}$. The idea is now that solving $\Delta_{2}D_{2}=Q_{2}$ is easier than solving $\Delta_{1}D_{1}=Q_{1}$, since the projection algorithm will neutralize some difficult eigenvalues of the iteration matrix. More specifically, we now set $\Delta_{2}=M_{2}-W_{2}$ where $M_{2}$ is diagonal and ${\left(M_{2}\right)}_{kk}={\left(\Delta_{2}\right)}_{kk}/\left(1-y_{k}-{\left(z_{2}\right)}_{k}\right)$, $T_{2}=M_{2}^{-1}W_{2}$, and $P_{2}=Q_{2}^{t}$, and we have the new convergent expansion [39]

(50) $$D^{\|}=\frac{u_{2}⊗u_{2}}{\langle u_{2},z_{2}\rangle }+\sum_{0\leq j<\infty }{\left(P_{2}T_{2}\right)}^{j}P_{2}M_{2}^{-1}P_{2}^{t}.$$

These new expansions, notably the two-term approximation $\frac{u_{2}⊗u_{2}}{\langle u_{2},z_{2}\rangle }+P_{2}M_{2}^{-1}P_{2}^{t}$, have been shown to give accurate results independently of the ionization degree [39].

We now consider the complex linear systems associated with the diffusion coefficients perpendicular and transverse to the magnetic field that are in the form for j∈S

(51) $$\left\{ \begin{array}{cc} \left(\Delta +i\Delta^{B}\right)\alpha^{D_{j}}= & \beta^{D_{j}},\\ \langle \alpha^{D_{j}},y\rangle = & 0, \end{array}\right.$$

for j∈S where $\Delta ,\Delta^{B}\in R^{n,n}$, $\alpha^{D_{j}}\in C^{n}$, and $\beta ,y\in R^{n}$. The matrix Δ and the constraint vector y are as in the isotropic case and $\Delta^{B}=QD^{\prime }P$ where $D^{\prime }$ is diagonal, Q=I−y⊗u and P=I−u⊗y, assuming naturally that 〈u,y〉=1. In particular, $N(\Delta +i\Delta^{B})=Cu$, the well-posedness property $N\left(\Delta +i\Delta^{B}\right)⊕y^{⊥}=C^{n}$ holds, and $\beta \in R(\Delta +i\Delta^{B})$. The iterative techniques already available for the isotropic systems have been extended to the magnetized situation, either for the generalized conjugate gradient type, or for the stationary type [28,29]. In particular, upon introducing the splitting $\Delta +i\Delta^{B}=M-W$, where $M=\mathrm{diag}\left(\Delta \right)+\mathrm{diag}\left(\sigma_{1},\ldots ,\sigma_{n}\right)+i\Delta^{B}$ is easily invertible, and the iteration matrix $T=M^{-1}W$, we have

$$D^{⊥}+iD^{⊙}=\sum_{0\leq j<\infty }{\left(PT\right)}^{j}PM^{-1}P^{t}.$$

The interest of these algorithms is that their convergence properties are always better in the magnetized case B≠0. Orthogonal residual algorithms may also be considered generalizing the use of conjugate gradients techniques already introduced for isotropic systems [39]. These algorithms may also be generalized to take into account increasing ionizations, as discussed for the matrix Δ, and we refer to the literature for more details [39]. Applications to expanding plasmas with specialized finite volume methods have also been developed by Peerenboom et al. [43,44].

## 4. Links Between the Kinetic Theories

We investigate in this section the links between the two different kinetic theories presented in the previous sections. The aim is to recover the structure of the multicomponent fluxes of the two-temperature kinetic theory in the special situation $T=T_{h}=T_{e}$ from that of the multicomponent fluxes of the one-temperature theory that does not take into account the smallness of the electron mass.

### 4.1. Recovering the Electron-to-Ion Mass Ratio

Our aim is to establish that the general structure of the two-temperature fluxes may be recovered, in the particular situation where $T=T_{h}=T_{e}$, from the fluxes of the one-temperature theory. In order to recover the structure of such nonequilibrium fluxes we will superimpose the scaling of the two-temperature theory within the transport linear systems of the one-temperature kinetic theory. For the sake of simplicity, only the first approximation of the diffusion velocities are again considered. The case of more general diffusion velocities resulting from larger Galerkin variational approximation spaces, the case of heat fluxes and thermal diffusion coefficients, and that of the viscous tensor may also be investigated by using using entirely similar methods mutatis mutandis. The fluid conservation equations of both models are then found to be asymptotically similar when both temperatures coincide.

The transport linear systems associated with the first approximation of the diffusion velocities have been obtained in Section 2.5. The first step is now to analyse the asymptotic behavior of the matrix Δ with the scaling of the two-temperature theory in order recover the smallness of the electron-to-ion mass ratio. The matrix Δ has been expressed in terms of the binary diffusion coefficients $D_{kl}^{\mathrm{bin}}$ that may be written

(52) $$D_{kl}^{\mathrm{bin}}=\frac{3}{16}\frac{\sqrt{2\pi {k_{\;B}}^{3}T^{3}/m_{kl}}}{p\pi \sigma_{kl}^{2}\Omega_{kl}^{(1,1)*}},$$

where $\sigma_{kl}$ denotes the collision diameter of the (k,l) species pair, $\Omega^{(1,1)*}$ a reduced collision integral, and $m_{kl}$ the reduced mass of the (k,l) species pair given by

(53) $$m_{kl}=\frac{m_{k}m_{l}}{m_{k}+m_{l}}.$$

These relations show that $D_{kl}^{\mathrm{bin}}=D_{kl}^{\mathrm{bin}}$ for k,l∈H, $D_{ek}^{\mathrm{bin}}=\epsilon \;D_{ek}^{\mathrm{bin}}$ for k∈H and $D_{ee}^{\mathrm{bin}}=\epsilon \;D_{ee}^{\mathrm{bin}}$ where $D_{kl}^{\mathrm{bin}}$ for k,l∈H, and $D_{ek}^{\mathrm{bin}}$ for k∈H are smooth functions of $\epsilon^{2}$, wheras $D_{ee}^{\mathrm{bin}}$ is a constant in ϵ. Moreover, since the Hall number of the heavy particles $\beta_{h}=q^{★}B^{★}t_{h}^{★}/m_{h}^{★}$ is such that $\beta_{h}=\epsilon \beta_{e}=\epsilon$, we deduce that $B=\epsilon \bar{B}$ where $\bar{B}$ is constant in ϵ.

We will freely use in the next sections the following result of linear algebra associated with generalized inverses where N(A) and R(A) denote the nullspace and range of a matrix *A*. For any linear spaces A and B that are in direct sum $A⊕B=R^{n}$, we also denote by $P_{A,B}$ the projector onto A along B.

**Proposition 1.** 
*Let $G\in R^{n,n}$ be a matrix, and let p and q be two linear subspaces of $\;R^{n}$ such that the direct sums $N\left(G\right)⊕p=R^{n}$ and $R\left(G\right)⊕q=R^{n}$ hold. Then, there exists a unique matrix Z such that GZG=G, ZGZ=Z, N(Z)=q, and R(Z)=p. The matrix Z is termed the generalized inverse of G with prescribed range p and nullspace q and Z is also such that $GZ=P_{R(G),q}$ and $ZG=P_{p,N(G)}$.*

This result also holds mutatis mutandis in $C^{n}$, that is, replacing the assumptions $N\left(G\right)⊕p=R^{n}$ and $R\left(G\right)⊕q=R^{n}$ by $N\left(G\right)⊕p=C^{n}$ and $R\left(G\right)⊕q=C^{n}$.

### 4.2. The Isotropic Diffusion Matrix

We investigate the transport linear systems associated with the first approximation of the multicomponent diffusion coefficients. We consider in this section the situation of isotropic transport linear systems that yields the isotropic multicomponent diffusion coefficients *D*, as well as the multicomponent diffusion coefficients parallel to the magnetic field $D^{\|}$ for magnetized flows, and the situation of nonisotropic multicomponent diffusion coefficients $D^{⊥}+iD^{⊙}$ is discussed in the next section.

The transport linear system associated with the multicomponent diffusion matrix is in the form $\Delta \;\alpha^{D_{j}}=\beta^{D_{j}}$, with the constraint $\langle \alpha^{D_{j}},y\rangle =0$ for j∈S, and $D_{ij}=\alpha_{i}^{D_{j}}=\alpha_{j}^{D_{i}}$ for i,j∈S. One may also define uniquely the matrix *D* as the unique generalized inverse of the matrix Δ with prescribed nullspace N(D)=Ry and range $R\left(D\right)=y^{⊥}$. The matrix *D* is symmetric positive semi-definite and such that ΔD=I−y⊗u and DΔ=I−u⊗y.

A first step is to analyse the asymptotic behavior of the matrix Δ by using the scaling of the two-temperature theory. With the scaling presented in Section 4.1, and from the relations expressing the binary diffusion coefficients, and that of the species masses, we deduce that $\Delta_{kl}={\bar{\Delta }}_{kl}$, k,l∈H, $\Delta_{ke}=\epsilon {\bar{\Delta }}_{ke}$, k∈H, $\Delta_{ek}=\epsilon {\bar{\Delta }}_{ek}$, k∈H, and $\Delta_{ee}=\epsilon {\bar{\Delta }}_{ee}$, where the coefficients ${\bar{\Delta }}_{kl}$, ${\bar{\Delta }}_{ke}$, and ${\bar{\Delta }}_{ee}$ are smooth positive functions of $\epsilon^{2}$. We may thus write

(54) $$\Delta =\left(\begin{array}{cc} \Delta_{hh} & \epsilon {\bar{\Delta }}_{he}\\ \epsilon {\bar{\Delta }}_{eh} & \epsilon {\bar{\Delta }}_{ee} \end{array}\right),$$

where $\Delta_{hh}={\left(\Delta_{kl}\right)}_{k,l\in H}$, $\Delta_{he}={\left(\Delta_{ke}\right)}_{k\in H}$, $\Delta_{eh}={\left(\Delta_{el}\right)}_{l\in H}$, ${\bar{\Delta }}_{hh}=\left({\bar{\Delta }}_{kl}\right)$, ${\bar{\Delta }}_{he}=\left({\bar{\Delta }}_{ke}\right)$, and ${\bar{\Delta }}_{eh}=\left({\bar{\Delta }}_{el}\right)$. We now introduce the partially rescaled variant matrices

(55) $$\Delta^{L}=\left(\begin{array}{cc} \Delta_{hh} & {\bar{\Delta }}_{he}\\ \epsilon {\bar{\Delta }}_{eh} & {\bar{\Delta }}_{ee} \end{array}\right),\;\Delta^{R}=\left(\begin{array}{cc} \Delta_{hh} & \epsilon {\bar{\Delta }}_{he}\\ {\bar{\Delta }}_{eh} & {\bar{\Delta }}_{ee} \end{array}\right),\;M_{\epsilon }=\left(\begin{array}{cc} I_{h} & 0_{n_{h},1}\\ 0_{1,n_{h}} & \epsilon \end{array}\right),$$

that are such that

(56) $$\Delta =\Delta^{L}M_{\epsilon }=M_{\epsilon }\Delta^{R}.$$

The superscripts ^L^ and ^R^ are used as mnemonics for Left and Right pseudo inverses. Letting $\tilde{u}=M_{\epsilon }u={\left(1,\ldots ,1,\epsilon \right)}^{t}$ we then note that $N\left(\Delta^{L}\right)=R\tilde{u}$, $R\left(\Delta^{L}\right)=u^{⊥}$, $N(\Delta^{R})=Ru$, and $R\left(\Delta^{R}\right)={\tilde{u}}^{⊥}$. Noting also that $y_{h}={\bar{y}}_{h}$ and $y_{e}=\epsilon^{2}{\bar{y}}_{e}$ where ${\bar{y}}_{h}$ and ${\bar{y}}_{e}$ are smooth functions of $\epsilon^{2}$, we have $y={\left({\bar{y}}_{h},\epsilon^{2}{\bar{y}}_{e}\right)}^{t}$ and we may introduce $\tilde{y}$ such that $\tilde{y}={\left({\bar{y}}_{h},\epsilon {\bar{y}}_{e}\right)}^{t}$, $M_{\epsilon }\tilde{y}=y$ and we also have $\langle u,y\rangle =\langle \tilde{u},\tilde{y}\rangle =1$. We may then introduce the generalized inverse $D^{L}$ of $\Delta^{L}$ with nullspace $N(D^{L})=Ry$ and range $R\left(D^{L}\right)={\tilde{y}}^{⊥}$, as well as the generalized inverse $D^{R}$ of $\Delta^{R}$ with nullspace $N\left(D^{R}\right)=R\tilde{y}$ and range $R\left(D^{R}\right)=y^{⊥}$. From the properties $N\left(\Delta^{L}\right)=R\tilde{u}$, $R\left(\Delta^{L}\right)=u^{⊥}$, $N(\Delta^{R})=Ru$, $R\left(\Delta^{R}\right)={\tilde{u}}^{⊥}$, 〈u,y〉=1, $\langle \tilde{u},\tilde{y}\rangle =1$, and the smoothness of generalized inverses, we deduce that both $D^{L}$ and $\Delta^{R}$ are smooth functions of ϵ over [0,1].

Now for ϵ positive, that is ϵ∈(0,1], we deduce from $\Delta =\Delta^{L}M_{\epsilon }$ and $\Delta =M_{\epsilon }\Delta^{R}$ that

(57) $$D^{L}=M_{\epsilon }D,\;D^{R}=D\;M_{\epsilon },$$

in such a way that $D_{h,h}=D_{h,h}^{R}=D_{h,h}^{L}$, $D_{h,e}=D_{h,e}^{L}$, $D_{e,h}=D_{e,h}^{R}$, and $\epsilon D_{e,e}=\epsilon D_{e,e}^{L}=\epsilon D_{e,e}^{R}$. We have thus recovered that the electron coefficient $D_{e,e}$ is of order O(1/ϵ), and that $D_{h,h}$, $D_{h,e}$, $D_{e,h}$ and $\epsilon D_{e,e}$ are smooth functions of ϵ. From the general relations $\Delta^{L}D^{L}=I-y⊗u$, we next obtain that

(58) $$\begin{array}{cc} \; & \Delta_{hh}D_{hh}+\epsilon {\bar{\Delta }}_{he}D_{eh}=I_{h}-y_{h}⊗u_{h}, \end{array}$$

(59) $$\begin{array}{cc} \; & \Delta_{hh}D_{he}+{\bar{\Delta }}_{he}\epsilon D_{ee}=-y_{h}⊗\left(1\right), \end{array}$$

(60) $$\begin{array}{cc} \; & {\bar{\Delta }}_{eh}D_{hh}+{\bar{\Delta }}_{ee}D_{eh}=-\epsilon {\bar{y}}_{\epsilon }⊗u_{h}, \end{array}$$

(61) $$\begin{array}{cc} \; & \epsilon {\bar{\Delta }}_{eh}D_{he}+{\bar{\Delta }}_{ee}\epsilon D_{ee}=1-\epsilon^{2}{\bar{y}}_{\epsilon }, \end{array}$$

where the third relation has been divided by ϵ. Similarly, from $D^{L}y=0$, we obtain that

(62) $$\begin{array}{cc} \; & D_{hh}y_{h}+\epsilon^{2}D_{he}{\bar{y}}_{e}=0, \end{array}$$

(63) $$\begin{array}{cc} \; & D_{eh}y_{h}+\epsilon^{2}D_{ee}{\bar{y}}_{e}=0, \end{array}$$

where the last relation has been divided by ϵ. Letting ϵ=0 in (58) and (62), we first obtain $\Delta_{hh}^{0}D_{hh}^{0}=I_{h}-y_{h}^{0}⊗u_{h}$ and $D_{hh}^{0}y_{h}^{0}=0$, where we have denoted $D_{hh}\left(0\right)$ by $D_{hh}^{0}$ and $\Delta_{hh}\left(0\right)$ by $\Delta_{hh}^{0}$, so that $D_{hh}^{0}$ is the generalized inverse of $\Delta_{hh}^{0}$ with nullspace $N\left(D_{hh}^{0}\right)=Ry_{h}^{0}$ and range $R\left(D_{hh}^{0}\right)={\left(y_{h}^{0}\right)}^{⊥}$. Note also that for ϵ=0, we have $\langle y_{h}^{0},u_{h}\rangle =1$. The heavy species thus do not see the electrons for diffusion when ϵ=0 anymore. Since $D_{hh}$ is a smooth function of ϵ, we also deduce that $D_{hh}=D_{hh}^{0}+O\left(\epsilon \right)$.

Considering then the electron diffusion coefficients, we first obtain from (61) that the limiting positive value of $\epsilon D_{ee}$ is $1/\;\Delta_{ee}^{0}$. We also note from (52) and (53) that ${\bar{\Delta }}_{ee}={\bar{\Delta }}_{ee}^{0}+O\left(\epsilon^{2}\right)$. From the relation (61), we next obtain that

$$D_{ee}=\frac{1}{\epsilon {\bar{\Delta }}_{ee}^{0}}-\frac{{\bar{\Delta }}_{eh}^{0}}{{\bar{\Delta }}_{ee}^{0}}D_{he}^{0}+O\left(\epsilon \right).$$

Letting ϵ=0 in the relation (60), we obtain that ${\bar{\Delta }}_{eh}^{0}D_{hh}^{0}=-{\bar{\Delta }}_{ee}^{0}D_{eh}^{0}$, and transposing this relation yields $D_{he}^{0}=-D_{hh}^{0}{\bar{\Delta }}_{he}^{0}/{\bar{\Delta }}_{ee}^{0}$, so that

(64) $$D_{ee}=\frac{1}{\epsilon {\bar{\Delta }}_{ee}^{0}}+\frac{{\bar{\Delta }}_{eh}^{0}}{{\bar{\Delta }}_{ee}^{0}}D_{hh}^{0}\frac{{\bar{\Delta }}_{he}^{0}}{{\bar{\Delta }}_{ee}^{0}}+O\left(\epsilon \right).$$

Considering finally the diffusion velocities parallel to the magnetic field $V^{\|}=-D^{\|}d^{\|}$, and noting that $D=D^{\|}$, we obtain that

(65) $$\begin{array}{cc} \; & V_{h}^{\|}=-D_{hh}^{0}d_{h}^{\|}-D_{he}^{0}d_{e}^{\|}+O\left(\epsilon \right) \end{array}$$

(66) $$\begin{array}{cc} \; & V_{e}^{\|}=-\left(\frac{1}{\epsilon {\bar{\Delta }}_{ee}^{0}}+\frac{{\bar{\Delta }}_{eh}^{0}}{{\bar{\Delta }}_{ee}^{0}}D_{hh}^{0}\frac{{\bar{\Delta }}_{he}^{0}}{{\bar{\Delta }}_{ee}^{0}}\right)d_{e}^{\|}+\frac{{\bar{\Delta }}_{eh}^{0}}{{\bar{\Delta }}_{ee}^{0}}D_{hh}^{0}d_{h}^{\|}+O\left(\epsilon \right), \end{array}$$

so that

$$V_{e}^{\|}=-\frac{1}{\epsilon \Delta_{ee}^{0}}d_{e}^{\|}+\frac{\Delta_{eh}^{0}}{\Delta_{ee}^{0}}V_{h}^{\|}+O\left(\epsilon \right).$$

This notably yields the second-order corrector diffusion driving force $-V_{h}^{\|}$ appearing in the electron diffusion velocity parallel to the magnetic field $V_{e}^{\|}$. We have thus recovered the structure of the two-temperature diffusion velocities with (65) and (66) from the one-temperature theory in the special situation where $T=T_{e}=T_{h}$. The second-order diffusion driving forces $V_{h}^{\|}$ of the two-temperature kinetic theory also naturally arise through the cross-diffusive terms of the one-temperature kinetic theory. The same asymptotic analysis may also be conducted with the Stefan–Maxwell equations as well as with larger transport linear systems that yield more accurate transport coefficients. All the transport linear systems may indeed be rewritten by separating the heavy species from the electrons with a scaling by blocks similar to that of the Δ matrix.

### 4.3. The Nonisotropic Diffusion Matrices

We now investigate the transport linear systems associated with the first approximation of the diffusion velocities in the magnetized situation. All other transport linear systems associated with larger variational approximations or other nonisotropic transport coefficients may indeed be investigated in a similar way.

The magnetized transport linear systems associated with the first approximation of the multicomponent diffusion matrices are in the form $\Delta \;\alpha^{D_{j}\left(1\right)}=\beta^{D_{j}}$ and $\left(\Delta +i\Delta^{B}\right)\alpha^{D_{j}\left(2\right)}=\beta^{D_{j}}$ with the constraints $\langle \alpha^{D_{j}\left(1\right)},y\rangle =0$ and $\langle \alpha^{D_{j}\left(2\right)},y\rangle =0$. The coefficients of the matrix Δ are given in the previous section and the coefficients of the matrix $\Delta^{B}$ are given by $\Delta^{B}=\left(I-y⊗u\right)\;D^{B}\left(I-u⊗y\right)$ where $D^{B}$ is the diagonal matrix $D_{kl}^{B}=\delta_{kl}n_{k}q_{k}B/p$, $y={\left(y_{1},\ldots ,y_{n}\right)}^{t}\in R^{n}$, and $u={\left(1,\ldots ,1\right)}^{t}\in R^{n}$. We discuss in this section the magnetized systems associated with the $\alpha^{D_{j}\left(2\right)}$ unknowns and the $D^{⊥}+iD^{⊙}$ matrix.

The complex matrix Δ+iΔ is such that $N(\Delta +i\Delta^{B})=Cu$, $R\left(\Delta +i\Delta^{B}\right)=u^{⊥}$ in $C^{n}$. The resulting diffusion coefficients $D^{⊥}$ and $D^{⊙}$ are then given by $D_{ij}^{⊥}+iD_{ij}^{⊙}=\langle \alpha^{D_{j}\left(2\right)},\beta^{D_{i}}\rangle =\alpha_{i}^{D_{j}\left(2\right)}=\alpha_{j}^{D_{i}\left(2\right)}$. The matrix $D^{⊥}+iD^{⊙}$ is the generalized inverse of $\Delta +i\Delta^{B}$ with prescribed nullspace $N(D^{⊥}+iD^{⊙})=Cy$ and range $R\left(D^{⊥}+iD^{⊙}\right)=y^{⊥}$ and we have the relations $\left(\Delta +i\Delta^{B}\right)\left(D^{⊥}+iD^{⊙}\right)=I-y⊗u$ and $\left(D^{⊥}+iD^{⊙}\right)\left(\Delta +i\Delta^{B}\right)=I-u⊗y$. We also have for any a>0 the relation $D^{⊥}+iD^{⊙}={\left(\Delta +i\Delta^{B}+ay⊗y\right)}^{-1}\;-\;\left(1/a\right)u⊗u$.

Then, using the scaling presented in Section 4.1 and from the relations expressing the binary diffusion coefficients, the species masses, and the magnetic field, we deduce that $\Delta_{kl}$ has the same scaling properties as in the previous section, whereas $\Delta_{kl}^{B}=\epsilon {\bar{\Delta }}_{kl}^{B}$, k,l∈S, where the coefficients ${\bar{\Delta }}_{kl}^{B}$ are smooth functions of $\epsilon^{2}$. The Hall number of the heavy particles $\beta_{h}$ is indeed such that $\beta_{h}=\epsilon \beta_{e}$, so that B is O(ϵ). We may thus write

(67) $$\tilde{\Delta }=\Delta +i\Delta^{B}=\left(\begin{array}{cc} \Delta_{hh} & \epsilon {\bar{\Delta }}_{he}\\ \epsilon {\bar{\Delta }}_{eh} & \epsilon {\bar{\Delta }}_{ee} \end{array}\right)+\epsilon i\left(\begin{array}{cc} {\bar{\Delta }}_{hh}^{B} & {\bar{\Delta }}_{he}^{B}\\ {\bar{\Delta }}_{eh}^{B} & {\bar{\Delta }}_{ee}^{B} \end{array}\right)=\left(\begin{array}{cc} \Delta_{hh}+\epsilon i{\bar{\Delta }}_{hh}^{B} & \epsilon {\bar{\Delta }}_{he}+\epsilon i{\bar{\Delta }}_{he}^{B}\\ \epsilon {\bar{\Delta }}_{eh}+\epsilon i{\bar{\Delta }}_{eh}^{B} & \epsilon {\bar{\Delta }}_{ee}+\epsilon i{\bar{\Delta }}_{ee}^{B} \end{array}\right),$$

with a block decomposition between heavy species and electrons.

We introduce the partially rescaled matrices

(68) $${\tilde{\Delta }}^{L}=\left(\begin{array}{cc} \Delta_{hh}+\epsilon i{\bar{\Delta }}_{hh}^{B} & {\bar{\Delta }}_{he}+i{\bar{\Delta }}_{he}^{B}\\ \epsilon {\bar{\Delta }}_{eh}+\epsilon i{\bar{\Delta }}_{eh}^{B} & {\bar{\Delta }}_{ee}+i{\bar{\Delta }}_{ee}^{B} \end{array}\right),\;{\tilde{\Delta }}^{R}=\left(\begin{array}{cc} \Delta_{hh}+\epsilon i{\bar{\Delta }}_{hh}^{B} & \epsilon {\bar{\Delta }}_{he}+\epsilon i{\bar{\Delta }}_{he}^{B}\\ {\bar{\Delta }}_{eh}+i{\bar{\Delta }}_{eh}^{B} & {\bar{\Delta }}_{ee}+i{\bar{\Delta }}_{ee}^{B} \end{array}\right),$$

that are such that

(69) $$\tilde{\Delta }=\Delta +i\Delta^{B}=M_{\epsilon }{\tilde{\Delta }}^{R}={\tilde{\Delta }}^{L}\;M_{\epsilon }.$$

Letting $\tilde{u}=Mu={\left(1,\ldots ,1,\epsilon \right)}^{t}$ and $\tilde{y}={\left(y_{h},\epsilon {\bar{y}}_{\epsilon }\right)}^{t}$, we then note that $N\left({\tilde{\Delta }}^{L}\right)=C\tilde{u}$, $R\left({\tilde{\Delta }}^{L}\right)=u^{⊥}$, $N({\tilde{\Delta }}^{R})=Cu$, $R\left({\tilde{\Delta }}^{R}\right)={\tilde{u}}^{⊥}$, and $\langle u,y\rangle =\langle \tilde{u},\tilde{y}\rangle =1$. We may then introduce the generalized inverse ${\tilde{D}}^{L}$ of ${\tilde{\Delta }}^{L}$ with nullspace $N({\tilde{D}}^{L})=Cy$ and range $R\left({\tilde{D}}^{L}\right)={\tilde{y}}^{⊥}$, as well as the generalized inverse ${\tilde{D}}^{R}$ of ${\tilde{\Delta }}^{R}$ with nullspace $N\left({\tilde{D}}^{R}\right)=C\tilde{y}$ and range $R\left({\tilde{D}}^{R}\right)=y^{⊥}$, and both ${\tilde{D}}^{L}$ and ${\tilde{D}}^{R}$ are smooth functions of ϵ over [0,1].

Denoting by $\tilde{D}$ the combined matrix $\tilde{D}=D^{⊥}+iD^{⊙}$, and since by definition ${\tilde{\Delta }}^{L}$ and ${\tilde{\Delta }}^{R}$ are simply rescaled variants of $\tilde{\Delta }$ with $\tilde{\Delta }={\tilde{\Delta }}^{L}M_{\epsilon }$ and $\tilde{\Delta }=M_{\epsilon }\;{\tilde{\Delta }}^{R}$, we directly obtain that for ϵ positive in (0,1], we have

(70) $${\tilde{D}}^{L}=M_{\epsilon }\tilde{D},\;{\tilde{D}}^{R}=\tilde{D}\;M_{\epsilon }.$$

We have thus established the properties ${\tilde{D}}_{h,h}={\tilde{D}}_{h,h}^{R}={\tilde{D}}_{h,h}^{L}$, ${\tilde{D}}_{h,e}={\tilde{D}}_{h,e}^{L}$, ${\tilde{D}}_{e,h}={\tilde{D}}_{e,h}^{R}$, and $\epsilon {\tilde{D}}_{e,e}=\epsilon {\tilde{D}}_{e,e}^{L}=\epsilon {\tilde{D}}_{e,e}^{R}$. This shows that all the matrices $D_{h,h}^{⊥}$, $D_{h,h}^{⊙}$, $D_{h,e}^{⊥}$, $D_{h,e}^{⊙}$, $D_{e,h}^{⊥}$, $D_{e,h}^{⊙}$, $\epsilon D_{e,e}^{⊥}$, and $\epsilon D_{e,e}^{⊙}$ are smooth functions of ϵ.

From the general relation between generalized inverses ${\tilde{\Delta }}^{L}{\tilde{D}}^{L}=I-y⊗u$, we obtain that

(71) $$\begin{array}{cc} \; & \left({\bar{\Delta }}_{hh}+i\epsilon \Delta_{hh}^{B}\right)\left(D_{hh}^{⊥}+D_{hh}^{⊙}\right)+\epsilon \left({\bar{\Delta }}_{he}+i{\bar{\Delta }}_{he}^{B}\right)\left(D_{eh}^{⊥}+iD_{eh}^{⊙}\right)=I_{h}-y_{h}⊗u_{h}, \end{array}$$

(72) $$\begin{array}{cc} \; & \left(\Delta_{hh}+i\epsilon {\bar{\Delta }}_{hh}^{B}\right)\left(D_{he}^{⊥}+D_{he}^{⊙}\right)+\left({\bar{\Delta }}_{he}+i{\bar{\Delta }}_{he}^{B}\right)\epsilon \left(D_{ee}^{⊥}+iD_{ee}^{⊙}\right)=-y_{h}⊗\left(1\right), \end{array}$$

(73) $$\begin{array}{cc} \; & \left({\bar{\Delta }}_{eh}+i{\bar{\Delta }}_{eh}^{B}\right)\left(D_{hh}^{⊥}+iD_{hh}^{⊙}\right)+\left({\bar{\Delta }}_{ee}+i{\bar{\Delta }}_{ee}^{B}\right)\left(D_{eh}^{⊥}+iD_{eh}^{⊙}\right)=-\epsilon {\bar{y}}_{\epsilon }⊗u_{h}, \end{array}$$

(74) $$\begin{array}{cc} \; & \epsilon \left({\bar{\Delta }}_{eh}+i{\bar{\Delta }}_{eh}^{B}\right)\left(D_{he}^{⊥}+iD_{he}^{⊙}\right)+\left({\bar{\Delta }}_{ee}+i{\bar{\Delta }}_{ee}^{B}\right)\epsilon \left(D_{ee}^{⊥}+iD_{ee}^{⊙}\right)=1-\epsilon^{2}{\bar{y}}_{\epsilon }, \end{array}$$

where the third relation has been divided by ϵ. Moreover, we also obtain from ${\tilde{D}}^{L}y=0$ that

(75) $$\begin{array}{cc} \; & \left(D_{hh}^{⊥}+iD_{hh}^{⊙}\right)y_{h}+\epsilon^{2}\left(D_{he}^{⊥}+iD_{he}^{⊙}\right){\bar{y}}_{e}=0, \end{array}$$

(76) $$\begin{array}{cc} \; & \left(D_{eh}+iD_{eh}^{⊙}\right)y_{h}+\epsilon^{2}\left(D_{ee}^{⊥}+iD_{ee}^{⊙}\right){\bar{y}}_{e}=0, \end{array}$$

where the last relation has been divided by ϵ.

Letting ϵ=0 in (71) and (75), we first obtain $\Delta_{hh}^{0}{\left(D_{hh}+iD_{hh}\right)}^{0}=I_{h}-y_{h}^{0}⊗u_{h}$ and ${\left(D_{hh}^{⊥}+iD_{hh}^{⊙}\right)}^{0}y_{h}^{0}=0$, where we have denoted $D_{hh}^{⊥}\left(0\right)+iD_{hh}^{⊙}\left(0\right)$ by ${\left(D_{hh}^{⊥}+iD_{hh}^{⊙}\right)}^{0}$ and $\Delta_{hh}\left(0\right)$ by $\Delta_{hh}^{0}$, so that ${\left(D_{hh}^{⊥}+iD_{hh}^{⊙}\right)}^{0}$ is the generalized inverse of $\Delta_{hh}^{0}$ with nullspace $N\left(D_{hh}^{0}\right)=Cy_{h}$ and range $R\left(D_{hh}^{0}\right)=y_{h}^{⊥}$. We thus deduce that $D_{hh}^{⊙}\left(0\right)=0$ from the uniqueness of generalized inverses so that $D_{hh}^{⊥}\left(0\right)$ coincide with the traditional diffusion matrix obtained without magnetic field $D_{hh}\left(0\right)=D_{hh}^{\|}\left(0\right)$. The heavy species thus do not see the electrons during their diffusive process anymore, and diffusion is not magnetized anymore. Since $D_{hh}$ is a smooth function of ϵ, we also deduce that $D_{hh}^{⊥}+iD_{hh}^{⊙}=D_{hh}^{0}+O\left(\epsilon \right)$.

Considering then the electron diffusion coefficients, we first obtain that the limiting positive value of $\epsilon D_{ee}$ is $1/\left({\bar{\Delta }}_{ee}+i{\bar{\Delta }}_{ee}^{B}\right)$. We also note that ${\bar{\Delta }}_{ee}+i{\bar{\Delta }}_{ee}^{B}={\left({\bar{\Delta }}_{ee}+i{\bar{\Delta }}_{ee}^{B}\right)}^{0}+O\left(\epsilon^{2}\right)$. From the relation (61), we next obtain that

$$D_{ee}^{⊥}+iD_{ee}^{⊙}=\frac{1}{\epsilon \left({\bar{\Delta }}_{ee}+i{\bar{\Delta }}_{ee}^{B}\right)}-\frac{{\left({\bar{\Delta }}_{ee}+i{\bar{\Delta }}_{ee}^{B}\right)}^{0}}{{\left({\bar{\Delta }}_{eh}+i{\bar{\Delta }}_{eh}^{B}\right)}^{0}}{\left(D_{he}+iD_{he}^{B}\right)}^{0}+O\left(\epsilon \right).$$

Letting ϵ=0 in the relation (60), we next obtain that

$${\left({\bar{\Delta }}_{eh}+i{\bar{\Delta }}_{eh}^{B}\right)}^{0}D_{hh}^{0}=-{\left({\bar{\Delta }}_{ee}+i{\bar{\Delta }}_{ee}^{B}\right)}^{0}{\left(D_{eh}+iD_{eh}^{B}\right)}^{0},$$

and transposing, we finally obtain that ${\left(D_{he}+iD_{he}^{B}\right)}^{0}=-D_{hh}^{0}{\left({\bar{\Delta }}_{he}+i{\bar{\Delta }}_{he}^{B}\right)}^{0}/{\left({\bar{\Delta }}_{ee}+i{\bar{\Delta }}_{ee}^{B}\right)}^{0}$, so that finally,

(77) $$D_{ee}^{⊥}+iD_{ee}^{⊙}=\frac{1}{\epsilon \left({\bar{\Delta }}_{ee}+i{\bar{\Delta }}_{ee}^{B}\right)}-\frac{{\left({\bar{\Delta }}_{ee}+i{\bar{\Delta }}_{ee}^{B}\right)}^{0}}{{\left({\bar{\Delta }}_{eh}+i{\bar{\Delta }}_{eh}^{B}\right)}^{0}}D_{hh}^{0}\frac{{\left({\bar{\Delta }}_{ee}+i{\bar{\Delta }}_{ee}^{B}\right)}^{0}}{{\left({\bar{\Delta }}_{he}+i{\bar{\Delta }}_{he}^{B}\right)}^{0}}+O\left(\epsilon \right).$$

Considering finally the diffusion velocities and using the property that for any vectors x and u, a relation in the form $x^{⊥}=\alpha u^{⊥}+\beta u^{⊙}$ is equivalent to the complex relation $x^{⊥}-ix^{⊙}=\left(\alpha +i\beta \right)\left(u^{⊥}-u^{⊙}\right)$, we may write the diffusion velocities orthogonal to the magnetic field in the form

(78) $$\begin{array}{cc} V_{h}^{⊥}-iV_{h}^{⊙}= & -D_{hh}^{0}\left(d_{h}^{⊥}-id_{h}^{⊙}\right)-{\left(D_{he}^{⊥}+iD_{he}^{⊙}\right)}^{0}\left(d_{e}^{⊥}-id_{e}^{⊙}\right)+O\left(\epsilon \right) \end{array}$$

(79) $$\begin{array}{cc} V_{e}^{⊥}-iV_{e}^{⊙}= & -\left(\frac{1}{\epsilon \left({\bar{\Delta }}_{ee}+i{\bar{\Delta }}_{ee}^{B}\right)}+\frac{{\left(\Delta_{ee}+i\Delta_{ee}^{B}\right)}^{0}}{{\left({\bar{\Delta }}_{eh}+i{\bar{\Delta }}_{eh}^{B}\right)}^{0}}D_{hh}^{0}\frac{{\left({\bar{\Delta }}_{ee}+i{\bar{\Delta }}_{ee}^{B}\right)}^{0}}{{\left({\bar{\Delta }}_{he}+i{\bar{\Delta }}_{he}^{B}\right)}^{0}}\right)\left(d_{e}^{⊥}-id_{e}^{⊙}\right) \end{array}$$

(80) $$\begin{array}{cc} \; & +\frac{{\left(\Delta_{ee}+i\Delta_{ee}^{B}\right)}^{0}}{{\left(\Delta_{eh}+i\Delta_{eh}^{B}\right)}^{0}}D_{hh}^{0}\left(d_{h}^{⊥}-id_{h}^{⊙}\right)+O\left(\epsilon \right), \end{array}$$

so that

$$V_{e}^{⊥}-iV_{e}^{⊙}=-\frac{1}{\epsilon \left({\bar{\Delta }}_{ee}+i{\bar{\Delta }}_{ee}^{B}\right)}\left(d_{e}^{⊥}-id_{e}^{⊙}\right)+\frac{{\left({\bar{\Delta }}_{ee}+i{\bar{\Delta }}_{ee}^{B}\right)}^{0}}{{\left({\bar{\Delta }}_{eh}+i{\bar{\Delta }}_{eh}^{B}\right)}^{0}}\left(V_{h}^{⊥}-iV_{h}^{⊙}\right)+O\left(\epsilon \right).$$

Taking the real part of this identity then yields the second-order corrector diffusion driving forces $V_{h}^{⊥}$ and $V_{h}^{⊙}$ appearing in the electron diffusion velocity $V_{e}^{⊥}$ in the magnetized situation. We have thus finally recovered the structure of the two-temperature diffusion velocities from the one-temperature theory in the special situation where $T=T_{e}=T_{h}$. The second-order correctors of the two-temperature kinetic theory also naturally arise through the cross-diffusive terms of the one-temperature kinetic theory. We have finally recovered the general structure of the two-temperature diffusion coefficients with the O(1/ϵ) contributions as well as a O(1) corrections.

## 5. Conclusions and Future Work

The two-temperature and one-temperature kinetic theories of reactive magnetized multicomponent plasmas with polyatomic species have been summarized. New links have been obtained between these theories by investigating the transport linear systems. Only strong magnetic fields have been considered in this paper, but the method is easily adapted to the situation of weak magnetic fields mutatis mutandis. The new links reveal that, in the special situation where electron and heavy species temperatures coincide, the thermal plasma kinetic theory contains similar terms to the two-temperature theory when superimposing the small electron mass scaling. In other words, for thermal plasmas, the traditional kinetic theory and the kinetic theory using a small mass ratio yields asymptotically similar models. This also confirms that the second-order correctors required in the two-temperature kinetic theory are not Burnett-type terms but usual terms shifted to second order because of the scaling.

The resulting two-temperature fluid plasma model is important for the numerical simulation of plasma-enhanced chemical vapor deposition processes and may be used in particular to model the fabrication of solar cells or for the large area deposition of silicon thin films for electronic applications [1,45]. On the other hand, multicomponent fluids with nonisotropic diffusion properties in strong magnetic fields play a key role for radio propagation in the ionized upper layer of the atmosphere as well as for planetary or solar physics [4,24].

Investigating multitemperature models involving internal vibrational temperatures as well as electron and translational temperatures would be of high scientific interest. Coupling with radiation plays a key role in spatial plasmas and could also be investigated by adding photons as extra particles [46]. Similarly, investigating moment methods for plasmas and their related stabilized versions would also be of high scientific interest. Finally, the interaction of plasma with walls should also be investigated with kinetic models taking into account the wall’s atoms.

## Data Availability

No new data were created or analyzed in this study.

## Appendix A. Index of Notation

We generally denote by *i* the mixture species index, eventually another integer when discussing the transport linear systems, *t* the time, x the three-dimensional spatial coordinate, $\nabla ={\left(\partial_{1},\partial_{2},\partial_{3}\right)}^{t}$ the usual differential operator, $c_{i}$ the velocity of the particles of the *i*th species, $m_{i}$ the mass of the particle *i*th species, $q_{i}$ the particle charge of the *i*th species, $E_{iI}$ the internal energy of the *i*th species in the ith state, $a_{iI}$ the degeneracy of the ith state, $Q_{i}$ the indexing set of the quantum states *i*of the ith species, and $f_{i}$ the distribution function of the *i*th species. We denote by S={1,…,n} the species indexing set, *n* is the number of species, H the heavy species indexing set, and e the electron so that we may also write S=H∪{e}. The subscripts _e_ and _h_ are used to characterize quantities associated with electrons or heavy species, respectively.

We denote by $Z_{i}^{\mathrm{int}}=\sum_{I\in Q_{i}}a_{iI}\exp\left(-\frac{E_{iI}}{k_{\;B}T}\right)$ the partition function for the internal energy of the *i*th species at temperature *T*, $Z_{i}^{\mathrm{tr}}={\left(2\pi m_{i}k_{\;B}T/{h_{P}}^{2}\right)}^{3/2}$ the translational partition per unit volume at temperature *T*, and $Z_{i}=Z_{i}^{\mathrm{tr}}Z_{i}^{\mathrm{int}}$ the complete partition function. For a family of functions $\xi_{i}$, i∈S, we use the compact notation $\xi ={\left(\xi_{i}\right)}_{i\in S}$, and similarly $\xi_{h}={\left(\xi_{i}\right)}_{i\in H}$ for a family of functions associated with the heavy species.

We denote by $n_{i}$ the number density of the *i*th species, *T* the temperature, $T_{e}$ the electron temperature, $T_{h}$ the heavy species temperature, E the electric field, B the magnetic field, B=B/|B| the reduced magnetic field, v the fluid velocity, $E^{\prime }=E+v\wedge B$ the electric field in the mixture reference frame with a similar notation in the heavy species reference frame, $C_{i}=c_{i}-v$ the thermal speed of the particles of the *i*th species in the mixture reference frame with a similar notation in the heavy species reference frame, $J_{ij}$, i,j∈H∪{e}, the scattering collision operators, and $C_{i}$, i∈S, the reactive collision operators. We denote by *p* the pressure, E the energy per unit volume, ${\bar{E}}_{i}^{\mathrm{int}}$ the average energy of the *i*th species, S the entropy per unit volume and $g_{i}$ the Gibbs function of the *i*th species per unit mass.

We denote by x∧y the cross product between vectors x and y as Chapman and Cowling [24] and Flanders [47]. For any vector x, we denote by $x^{\|}=x\cdot \;B\;\;B$ $x^{⊥}=x-x^{\|}$, and $x^{⊙}=B\wedge x$ the associated vectors. These vectors $x^{\|}$, $x^{⊥}$ and $x^{⊙}$ are mutually orthogonal and obtained from x by applying the linear operators $M^{\|}=B⊗B$, $M^{⊥}=I-B⊗B$ and $M^{⊙}=R\left(B\right)$, where R(B) is the rotation operator associated with the vector B so that R(B)x=B∧x.

For macroscopic models, we generically denote by v the fluid velocity, $\rho_{i}=m_{i}n_{i}$ the mass density of the *i*th species, Q the heat flux, Π the viscous tensor, $V_{i}$ the diffusion velocity of the *i*th species, $\omega_{i}$ the molecular production of the *i*th species, $\rho =\sum_{i\in S}\rho_{i}$ the total mass density, p the pressure, Π the viscous tensor, nq the charge per unit volume, E the energy per unit volume, and J the conduction current.
